# Quantifying the impacts of drought and ecological restoration on net primary production changes in the Chinese Loess Plateau

**DOI:** 10.1371/journal.pone.0238997

**Published:** 2020-09-24

**Authors:** Xiaowei Jiang, Jianjun Bai

**Affiliations:** School of Geography and Tourism, Shaanxi Normal University, Xi’an, China; Shandong University, CHINA

## Abstract

Net primary production (NPP) can regulate global climate change and carbon balance. Although scholars have qualitatively studied the influencing factors of NPP, few have quantified the contribution of different degrees of drought aggravation or mitigation and major land-use changes to NPP changes. Based on the temporal and spatial characteristics of NPP for 2000–2015 in the Chinese Loess Plateau, we quantified the contribution of drought, land use and land cover change (LUCC), and hydrothermal conditions to changes in NPP. Particularly, we analyzed the contribution of major land-use change and different drought levels to NPP. Our results showed that the 15-years average NPP was approximately 227 gC/m^2^ and decreased from southeast to northwest. Overall, NPP showed a linear increasing trend over the 15-years period. The results suggested that changes in hydrothermal conditions had the strongest impact on NPP (~61%), followed by drought (~33%), and land use and land cover change had the weakest impact (~1.4%). In particular, ~13% of the NPP decreases was affected by light drought aggravation, ~10% of the NPP decreases was affected by moderate drought aggravation, and ~0.3% was affected by the conversion of cropland to grassland or non-NPP main production land. Moreover, ~12.7% of the NPP increase was affected by light drought alleviation, ~9.4% was affected by moderate drought alleviation, and ~1.1% was affected by the conversion of grassland to cropland or forestland. The mechanisms underlying the effect of drought and land-use change on NPP were clarified and provide an important reference value for future research on the carbon cycle and regional ecological environmental restoration.

## 1. Introduction

Since the industrial revolution, the massive combustion of fossil fuels has led to an increased release of greenhouse gases, such as CO_2_ [1, 2]. Subsequently, a series of environmental problems have occurred. As the first step of carbon sequestration in an ecosystem [3], net primary production (NPP) represents the increasing concentration of greenhouse gases, and plays an important role in regulating global climate change and the carbon balance, also, it is an important indicator for evaluating the sustainable development of terrestrial ecology [4]. Then, the influencing factors and driving mechanisms of NPP are important to understand. The academic community generally believes that climate factors and land use and land cover change (LUCC) are the main factors that influence NPP [5–7]. Cleveland et al. found that climate factors are the strongest predictors of NPP changes in tropical rain forests [6]. Dailiang et al. found that land-use change is one of the main factors affecting NPP [7]. The main factors underlying drought, such as precipitation, temperature and evapotranspiration, restrict the growth of vegetation and become the important factors for NPP [8,9]. Zhao et al. found that large-scale drought led to a decrease in global NPP [10]. In general, accurately evaluating the specific contributions of each influencing factor to changes in NPP is difficult. Furthermore, it is discovered that few studies have quantified the effect of different degrees of drought aggravation or mitigation and major land-use change to NPP changes [11–13].

The Loess Plateau is a typical arid and semiarid region in China that serves as an important ecological protection zone. The shortage of water resources makes the ecological environment of this region vulnerable and sensitive to climate change [14]. In the 1990s, the Chinese government adopted a series of ecological restoration policies to prevent and control the degradation of the ecological environment on the Loess Plateau, which have achieved some results [15]. Several studies have focused on evaluating the impact of climate change and anthropogenic activities on NPP in the Chinese Loess Plateau. For example, they [16–19] have found that NPP has generally shown an increasing trend with the alleviation of drought and the implementation of ecological measures over the past twenty years. Liu et al. analyzed the effects of vegetation types, topographic factors, climate change and human activities of the Loess Plateau grassland and found that precipitation and the Grain to Green Program promoted increased NPP [16]; taking Shaanxi Province of the Loess Plateau as an example, Wang et al. analyzed the relationship between evapotranspiration and NPP [17]; Shi et al. found that NPP on the Loess Plateau had a significant positive correlation with the precipitation from 1982–2014 [18]; Gang et al. studied the core area of the Grain to Green Program in the Loess Plateau and found that NPP increased significantly from 2000–2015 [19]; however, most of the studies were limited to a specific area of the Loess Plateau or a single vegetation type and single NPP influencing factors. For the entire Loess Plateau, whether the dominant factor of NPP change is drought or LUCC is unknown. In addition, few studies have quantified the effects of different levels of drought aggravation or mitigation and different land-use conversions on NPP changes in the Chinese Loess Plateau. Based on the spatial and temporal changes of NPP in the Loess Plateau from 2000 to 2015, we quantified the contribution of hydrothermal conditions, drought, and LUCC to the changes in NPP. Particularly, we analyzed the effects of major land-use changes and different levels of drought aggravation or mitigation on NPP. These results provide an important reference value for future research on the carbon cycle and regional ecological environmental restoration.

## 2. Data and methodology

### 2.1 Study area

**Fig 1:**
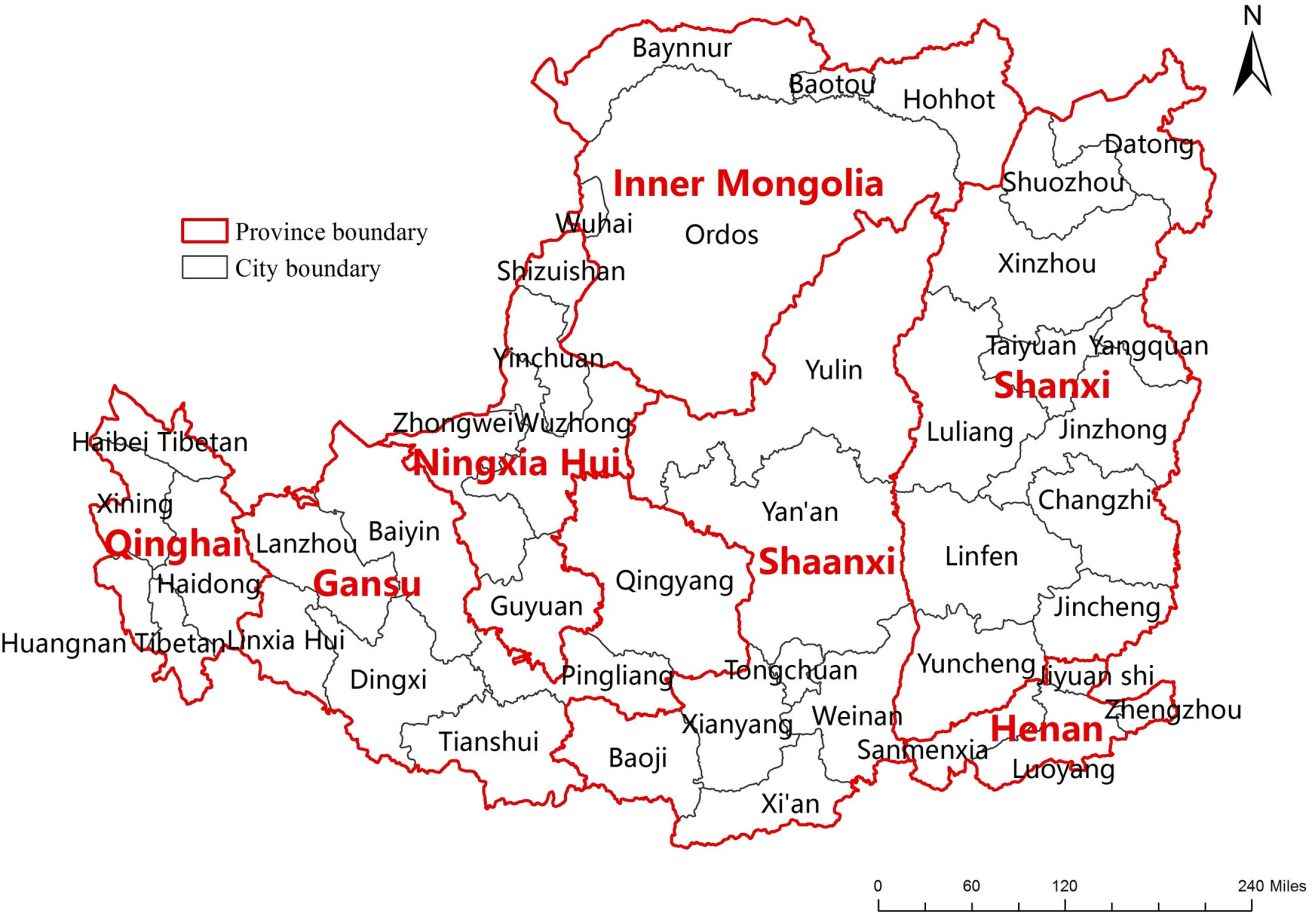
Provincial and prefecture-level administrative maps of the Loess Plateau.

**Fig 2:**
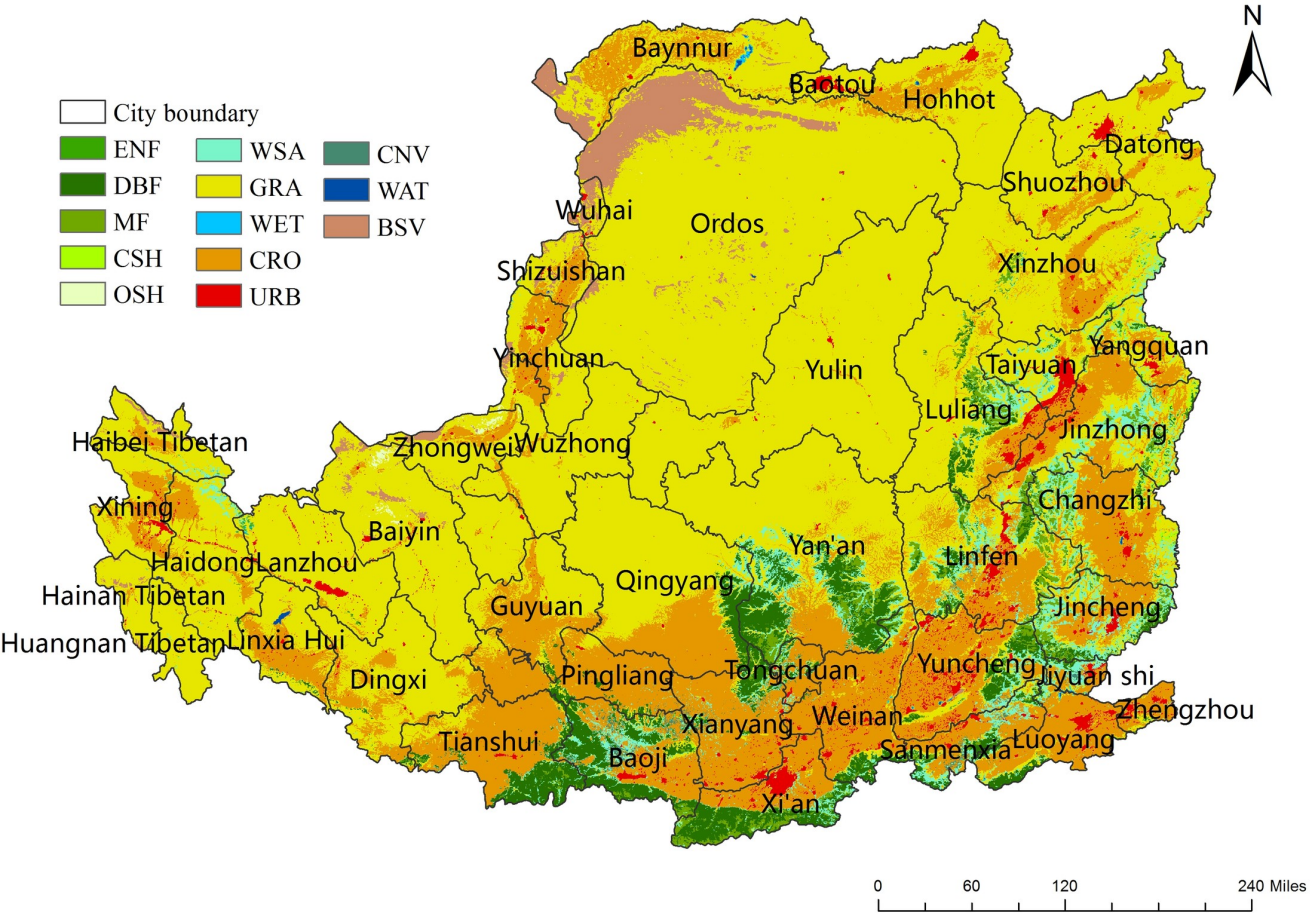
Land cover distribution derived from MCD12Q1 in 2010 for the Loess Plateau. Land cover types include evergreen needleleaf forest (ENF), deciduous broadleaf forest (DBF), mixed forest (MF), closed shrublands (CSH), open shrublands (OSH), woody savannas (WSA), grasslands (GRA), permanent wetlands (WET), croplands (CRO), urban and built-up (URB), cropland/natural vegetation mosaic (CNV), barren or sparsely vegetated (BSV), and water (WAT).

The Loess Plateau spans 44 cities in 7 provinces across the middle of the Yellow River in China, covering an area of 624,000 km^2^ (Fig 1). The plateau can be divided into three parts: east, middle, and west, with the Liupan Mountain and the Luliang Mountain as the boundary. The landforms of the Loess Plateau can be divided into plateau gullies, hilly gullies, mountains, loess gullies and river valley plains. The average annual temperature is 3.6~14.3°C, and the water resources are scarce [20]. The average annual precipitation is 150–750 mm, which is concentrated from July-September mostly in the form of rainstorms. Evaporation is generally higher than precipitation. According to the MODIS land cover product (MCD12Q1), grassland and cropland are the main land cover in this region. Grassland, cropland or forestland accounts for 61%, 23% and 10% of the total area, respectively (using 2011 as an example) (Fig 2).

### 2.2 Data collection and preprocessing

The NPP data (1 km, Alberts_1940) were derived from the Resource and Environmental Data Cloud Platform of the Chinese Academy of Sciences (http://www.resdc.cn/Default.aspx), which was calculated by the light energy utilization model GLM_PEM. GLM_PEM that used remote sensing to calculate vegetation productivity based on the absorption and utilization of photosynthetically active radiation [21].

Precipitation and temperature are the main climatic factors that affect NPP in arid and semiarid regions [22–24]. Compared with the standard precipitation index (SPI), the standardized precipitation evaporation index (SPEI) can reflect the impact of temperature on drought [25], and compared with the Palmer drought severity index (PDSI), SPEI had multiple time scales [26]. In this study, the SPEI came from the global monthly SPEI dataset with a time scale of 12 months and a spatial resolution of 0.5° (http://digital.csic.es). We resampled the SPEI data to the resolution and projection coordinate of the NPP data (1 km, Alberts_1940) and then analyzed the impact of drought on NPP.

Land cover/land use is a major factor that affects NPP [27, 28]. The MODIS land cover products (MCD12Q1, 500 m) for 2001 to 2015 (https://ladsweb.modaps.eosdis.nasa.gov) were used. The land cover scheme was provided by the International Geosphere-Biosphere Program (IGBP) land cover classification scheme. We resampled the MODIS data to the resolution and projection coordinate of the NPP data (1 km, Alberts_1940).

Because MODIS land cover products for 2000 were unavailable, we calculated the contribution of drought and LUCC to NPP in the Loess Plateau from 2001 to 2015.

### 2.3 Statistical analysis

First, regression analysis was used to analyze the linear trend of NPP and SPEI for 2000 to 2015. The linear change value *α* and the linear change rate *β* are shown in Eq 1 and Eq 2, respectively.

Second, regression analysis was used to calculate the correlation coefficient R of NPP and the SPEI-12 (Eq 3). Subsequently, the p-value was evaluated and the areas with p<0.05 were recorded as a significant correlation.

$$\alpha =\frac{\sum_{i=1}^{n}t_{i}x_{i}-\frac{1}{n}\sum_{i=1}^{n}t_{i}\sum_{i=1}^{n}x_{i}}{\sum_{i=1}^{n}{t_{i}}^{2}-\frac{1}{n}{(\sum_{i=1}^{n}t_{i})}^{2}}$$ (1)

$$\beta =\alpha /\bar{x}*16*100\%$$ (2)

$$R=\frac{\sum_{i=1}^{n}\left(NPP-\bar{NPP})(SPEI-\bar{SPEI}\right)}{\sqrt{\sum_{i=1}^{n}{\left(NPP-\bar{NPP}\right)}^{2}\sum_{i=1}^{n}{\left(SPEI-\bar{SPEI}\right)}^{2}}}$$ (3)

In the formulas, *α* is the linear change value; n is the number of years in the monitoring period, *n* = 16; i is the serial number of the year, *i* = 1,2,3…..16; *t*_*i*_ is the *i*-th year; x_i_ is the NPP value or SPEI value in year *i*; *β* is the linear change rate; $\bar{x}$ is the multiyear average value of NPP or SPEI; $(\bar{NPP)}$ is the multiyear average value of NPP; and $(\bar{SPEI)}$ is the multiyear average value of the SPEI.

### 2.4 Quantifying the contribution of drought, land use and land cover change (LUCC), and hydrothermal conditions to changes in NPP

According to the international standard for classifying drought grades based on the SPEI, droughts was classified as drought-free, light drought, moderate drought, severe drought and extreme drought (Table 1).

**Table 1:** Classification of drought grades based on the SPEI.

Drought grade	SPEI value
drought-free	SPEI>-0.5
light drought	-1<SPEI≤-0.5
moderate drought	-1.5<SPEI≤-1
severe drought	-2<SPEI≤-1.5
extreme drought	SPEI≤-2

SPEI>-0.5 was defined as normal or humid and SPEI≤-0.5 as drought or severe drought. The areas classified as drought or aggravated drought and normal or wet for two consecutive years were recorded as D (Eq 4) and N (Eq 5), respectively. The SPEI can reflect hydrothermal condition changes, and its decrease or increase was recorded as SPEI (d) (Eq 6) and SPEI (I) (Eq 7), respectively. In Eqs 4–7, i represents the year.

$$D=\left\{ \begin{array}{c} 1\;(({SPEI}_{i}>(-0.5)and\;{SPEI}_{i+1}\leq \left(-0.5\right))or\left({SPEI}_{i+1}<{SPEI}_{I}\leq (-0.5)\right))\\ 0\;or\;\mathrm{else} \end{array}\right.$$ (4)

$$N=\left\{ \begin{array}{c} 1\;(\left({SPEI}_{i}\leq (-0.5)and\;{SPEI}_{i+1}>(-0.5\right))or({SPEI}_{i}<{SPEI}_{i+1}\leq (-0.5)))\\ 0\;\mathrm{or}\;\mathrm{else} \end{array}\right.$$ (5)

$$SPEI\;(d)=\left\{ \begin{array}{c} {1\;SPEI}_{i+1}<{SPEI}_{i}\\ 0\;or\;\mathrm{else} \end{array}\right.$$ (6)

$$SPEI\;(I)=\left\{ \begin{array}{c} {1\;SPEI}_{i+1}>{SPEI}_{i}\\ 0\;or\;\mathrm{else} \end{array}\right.$$ (7)

**Fig 3:**
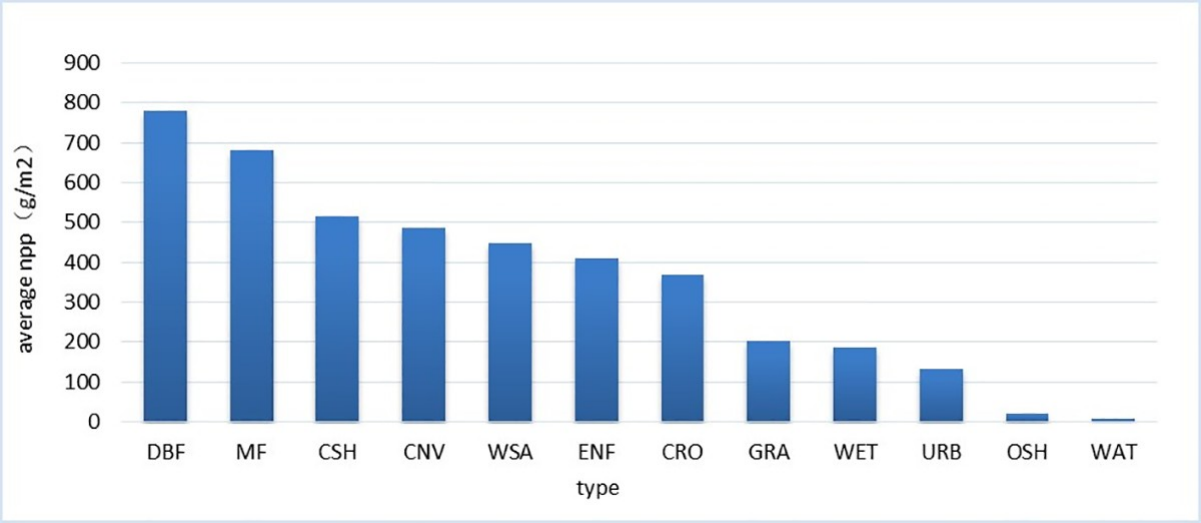
Average NPP (gC/m^2^) from different land-use types on the Loess Plateau from 2000 to 2015.

The study area was divided into 1 km × 1 km grids. Considering the ability of different land cover to generate NPP (Fig 3) and based on previous research [29, 30], we estimated the impact of land cover changes on NPP (Table 2). *R*_*_LCC(↓*)_ and *I*_*_LCC(↑)*_ indicate the area where land cover changes caused a decrease or increase in NPP, respectively (Eqs 8 and 9).

$$R_{\_LCC(↓)}=\{1\{\begin{array}{c} LCC(DBF,MF)to\;LCC(CSH,CNV,WSA,CRO,GRA)\\ or\;LCC(CSH,CNV,WSA,ENF,CRO)to\;LCC(GRA,WET,URB)\\ orLCC(GRA,WET)to\;LCC(WAT,OSH)\\ 0\;or\;else \end{array}$$ (8)

$$I_{\_LCC(↑)}=\{1\{\begin{array}{c} LCC(WAT,OSH)to\;LCC(GRA,WET)\\ or\;LCC(GRA,WET)to\;LCC(CSH,CNV,WSA,MF,ENF,CRO)\\ orLCC(GRA,CRO,WSA,CSH)to\;LCC(DBF,MF)\\ 0\;or\;else \end{array}$$ (9)

**Table 2:** Changes in NPP caused by land cover type conversion.

	DBF	MF	CSH	CNV	WSA	ENF	CRO	GRA	WET	URB	OSH	WAT
**DBF**	○	×	↓	↓	↓	×	↓	↓	×	×	×	×
**MF**	×	○	↓	↓	↓	×	↓	↓	×	×	×	×
**CSH**	↑	↑	○	○	○	○	○	↓	×	×	×	×
**CNV**	×	×	○	○	○	×	○	↓	×	↓	×	×
**WSA**	↑	↑	○	○	○	○	○	↓	↓	↓	×	×
**ENF**	×	×	○	○	○	○	○	↓	×	×	×	×
**CRO**	↑	↑	○	○	○	○	○	↓	↓	↓	×	×
**GRA**	↑	↑	↑	↑	↑	↑	↑	○	○	○	↓	↓
**WET**	×	↑	×	↑	↑	↑	↑	○	○	×	×	↓
**URB**	×	×	×	×	×	↑	×	×	×	○	×	×
**OSH**	×	×	×	×	×	×	×	↑	×	×	○	×
**WAT**	×	×	×	×	×	×	×	↑	↑	○	○	○

“↑” indicates that land cover change promotes increased NPP, “↓” indicates that land cover change promotes reduced NPP, “×” indicates an illogical result in terms of ground cover change or a scenario that will not happen in the short term, and “○” indicates that land cover change promotes little change in NPP.

The decrease and increase in NPP for two consecutive years were recorded as NPP (↓), i.e., *NPP*_*i + 1*_<*NPP*_*i*_ and NPP (↑), i.e., *NPP*_*i + 1*_>*NPP*_*i*_ (Eq 10), respectively. The total decrease or increase in NPP for 2001 to 2015 were recorded as *S*_*↓NPP*_ or *S*_*↑NPP*_, respectively (Eq 11). The total decrease or increase in NPP caused by drought aggravation or drought alleviation over 15 years were recorded as *S*_*↓D_NPP*_ and S_*↑D_NP*P_, respectively (Eq 12). The total decrease or increase in NPP caused by the overlapping area of drought and land cover change were recorded as *S*_*↓D_LCC_NPP*_ and *S*_*↑D_LCC_NPP*_, respectively (Eq 13). The total contribution of drought to the decrease or increase in NPP for 2001 to 2015 were recorded as *SCR*_*↓D_NPP*_ and *SCR*_*↑D_NPP*,_ respectively (Eq 14).

$$NPP_{↓↑}=\left\{ \begin{array}{l} NPP(↓)=↓({NPP}_{i+1}-{NPP}_{i}),\;({NPP}_{i+1}<{NPP}_{i})\\ NPP(↑)=↑({NPP}_{i+1}-{NPP}_{i}),\;({NPP}_{i+1}>{NPP}_{i}) \end{array}\right.$$ (10)

$$S_{↓↑(NPP)}=\left\{ \begin{array}{l} S_{↓NPP}=\sum_{i=2001}^{2015}\left(↓({NPP}_{i+1}-{NPP}_{i})),\;({NPP}_{i+1}<{NPP}_{i}\right)\\ S_{↑NPP}=\sum_{i=2001}^{2015}\left(↑({NPP}_{i+1}-{NPP}_{i})\right),\;({NPP}_{i+1}>{NPP}_{i}) \end{array}\right.$$ (11)

$$S_{↓↑(D\_NPP)}=\left\{ \begin{array}{l} S_{↓D\_NPP}=\sum_{i=2001}^{2015}\left(↓({NPP}_{i+1}-{NPP}_{i})*D),\;({NPP}_{i+1}<{NPP}_{i}\right)\\ S_{↑D\_NPP}=\sum_{i=2001}^{2015}(↑({NPP}_{i+1}-{NPP}_{i})*N),\;({NPP}_{i+1}>{NPP}_{i}) \end{array}\right.$$ (12)

$$S_{↓↑(D\_\_LCC\_NPP)}=\left\{ \begin{array}{l} S_{↓D\_LCC\_NPP}=\sum_{i=2001}^{2015}\left(↓({NPP}_{i+1}-{NPP}_{i})*D*R_{LCC(↓)}),\;({NPP}_{i+1}<{NPP}_{i}\right)\\ S_{↑D\_\_LCCNPP}=\sum_{i=2001}^{2015}\left(↑({NPP}_{i+1}-{NPP}_{i})*N*I_{LCC(↑)}\right),\;({NPP}_{i+1}>{NPP}_{i}) \end{array}\right.$$ (13)

$$SCR_{↓↑(D\_NPP)}=\left\{ \begin{array}{c} \frac{S_{↓D\_NPP}-S_{↓D\_LCC\_NPP}}{S_{↓NPP}}*100\%,\;({NPP}_{i+1}<{NPP}_{i})\\ \frac{S_{↑D\_NPP}-S_{↑D\_\_LCCNPP}}{S_{↑NPP}}*100\%,\;({NPP}_{i+1}>{NPP}_{i}) \end{array}\right.$$ (14)

Similarly, *S*_*↓LCC_NPP*_ and *S*_*↑LCC_NPP*_ indicate the total decrease or increase in NPP caused by land cover change, respectively (Eq 15); *SCR*_*↓LCC_NPP*_ and *SCR*_*↑LCC_NPP*_ indicate the total contribution of land cover change to the decrease or increase in NPP, respectively (Eq 16); *S*_*↓SPEI(d)_NPP*_ and *S*_*↑SPEI(I)_NPP*_ indicate the total decrease or increase in NPP caused by hydrothermal conditions, respectively (Eq 17). The total decrease or increase in NPP caused by the overlapping area of SPEI and land cover change was recorded as *S*_*↓SPEI_LCC_NPP*_ and *S*_*↑SPEI_LCC_NPP*_, respectively (Eq 18), and the total contribution of hydrothermal conditions to the decrease or increase in NPP is recorded as *SCR*_*↓SPEI_NPP*_ and *SCR*_*↑SPEI_NPP*_, respectively (Eq 19).

$$S_{↓↑(LCC\_NPP)}=\left\{ \begin{array}{l} S_{↓LCC\_NPP}=\sum_{i=2001}^{2015}\left(↓({NPP}_{i+1}-{NPP}_{i})*R_{LCC(↓)}),\;({NPP}_{i+1}<{NPP}_{i}\right)\\ S_{↑LCC\_NPP}=\sum_{i=2001}^{2015}(↑({NPP}_{i+1}-{NPP}_{i})*I_{LCC(↑)}),\;({NPP}_{i+1}>{NPP}_{i}) \end{array}\right.$$ (15)

$$SCR_{↓↑(LCC\_NPP)}=\left\{ \begin{array}{c} \frac{S_{↓LCC\_NPP}}{S_{↓NPP}}*100\%,\;({NPP}_{i+1}<{NPP}_{i})\\ \frac{S_{↑LCC\_NPP}}{S_{↑NPP}}*100\%,\;({NPP}_{i+1}>{NPP}_{i}) \end{array}\right.$$ (16)

$$S_{↓↑(SPEI\_NPP)=}\left\{ \begin{array}{c} S_{↓SPEI(d)\_NPP}=\sum_{i=2001}^{2015}\left(↓({NPP}_{i+1}-{NPP}_{i})*SPEI(d)),\;({NPP}_{i+1}<{NPP}_{i}\right)\\ S_{↑SPEI(I)\_NPP}=\sum_{i=2001}^{2015}(↑({NPP}_{i+1}-{NPP}_{i})*SPEI(I)),\;({NPP}_{i+1}>{NPP}_{i}) \end{array}\right.$$ (17)

$$S_{↓↑(SPEI\_\_LCC\_NPP)=}\left\{ \begin{array}{c} S_{↓SPEI\_LCC\_NPP}=\sum_{i=2001}^{2015}\left(↓({NPP}_{i+1}-{NPP}_{i})*SPEI(d)*R_{LCC(↓)}),\;({NPP}_{i+1}<{NPP}_{i}\right)\\ S_{↑SPEI\_LCC\_NPP}=\sum_{i=2001}^{2015}(↑({NPP}_{i+1}-{NPP}_{i})*SPEI(I)*I_{LCC(↑)}),\;({NPP}_{i+1}>{NPP}_{i}) \end{array}\right.$$ (18)

$$SCR_{↓↑(SPEI\_NPP)}=\left\{ \begin{array}{c} \frac{S_{↓SPEI\_NPP}-S_{↓SPEI\_LCC\_NPP}}{S_{↓NPP}}*100\%,\;({NPP}_{i+1}<{NPP}_{i})\\ \frac{S_{↑SPEI\_NPP}-S_{↑SPEI\_\_LCC\_NPP}}{S_{↑NPP}}*100\%,\;({NPP}_{i+1}>{NPP}_{i}) \end{array}\right.$$ (19)

### 2.5 Quantifying the contribution of major land-use change to NPP

According to China's land-use classification system, cropland (CRO) and cropland/natural vegetation mosaic (CNV) were merged into cropland; evergreen needleleaf forest (ENF), deciduous broadleaf forest (DBF), mixed forest (MF), closed shrublands (CSH), open shrublands (OSH) and woody savannas (WSA) were merged into forest; water (WAT) and permanent wetlands (WET) were merged into water area; and grasslands (GRA), urban and built-up (URB), and barren or sparsely vegetated (BSV) were defined as grassland, urban land, and unused land, respectively. The land-use transfer matrix of the Loess Plateau for 2001 to 2015 is shown in Table 3. The main land uses (cropland, forestland, and grassland) changed drastically and had the greatest impact on NPP. We focused on quantifying the contribution of these three land uses and non-NPP production land use (water area, urban land, unused land) to NPP change. *R*_*_LUCC (↓*)_ and *I*_*_LUCC (↑)*_ indicate the area where the land use change caused NPP decrease or increase, respectively (Eqs 20 and 21)); *S*_*↓LUCC_NPP*_ and *S*_*↑LUCC_NPP*_ (Eq 22) indicate the total decrease or increase in NPP caused by land use change, respectively; and *SCR*_*↓LUCC_NPP*_ and *SCR*_*↑LUCC_NPP*_ (Eq 23) indicate the total contribution of land use change to the decrease or increase in NPP, respectively.

$$R_{\_LUCC(↓)}=\{1\{\begin{array}{c} LUCC(FOR)to\;LUCC(CRO,GRA,CRI,WAT,UNU)\\ or\;LUCC(GRO)to\;LUCC(GRA,CRI,WAT,UNU)\\ orLUCC(GRA)to\;LUCC(CRI,WAT,UNU)\\ 0\;or\;else \end{array}$$ (20)

$$I_{\_LUCC(↑)}=\{1\{\begin{array}{c} LUCC(CRO)to\;LUCC(FOR)\\ or\;LUCC(GRA)to\;LUCC(CRO,FOR)\\ 0\;or\;else \end{array}$$ (21)

In Eqs 20 and 21, FOR, CRO, GRA, WAT, CRI, and UNU represent cropland, forest, grassland, water area, urban land, and unused land, respectively.

$$S_{↓↑(LUCC\_NPP)}=\left\{ \begin{array}{c} S_{↓LUCC\_NPP}=\sum_{i=2001}^{2015}\left(↓({NPP}_{i+1}-{NPP}_{i})*R_{LUCC(↓)}),\;({NPP}_{i+1}<{NPP}_{i}\right)\\ S_{↑LUCC\_NPP}=\sum_{i=2001}^{2015}(↑({NPP}_{i+1}-{NPP}_{i})*I_{LUCC(↓)}),\;({NPP}_{i+1}>{NPP}_{i}) \end{array}\right.$$ (22)

$$SCR_{↓↑(LUCC\_NPP)}=\left\{ \begin{array}{c} \frac{S_{↓LUCC\_NPP}}{S_{↓NPP}}*100\%,\;({NPP}_{i+1}<{NPP}_{i})\\ \frac{S_{↑LUCC\_NPP}}{S_{↑NPP}}*100\%,\;({NPP}_{i+1}>{NPP}_{i}) \end{array}\right.$$ (23)

**Table 3:** Land-use transfer matrix for 2001–2015 on the Loess Plateau (km^2^).

	urban land	cropland	forest	grassland	unused land	water
**urban land**	10564.3	619.0	130.3	265.3	14.7	0.7
**cropland**	270.3	121218.0	7207.6	24297.5	19.5	0.8
**forest**	15.1	4065.6	50849.6	12260.2	139.3	41.5
**grassland**	88.0	7006.2	3500.0	359925.0	5259.5	22.3
**unused land**	3.7	25.4	4.8	820.0	14360.8	13.4
**water**	0.2	37.4	144.1	202.1	43.4	447.3

### 2.6 Quantifying the contribution of different degrees of drought aggravation or mitigation to NPP changes

**Fig 4:**
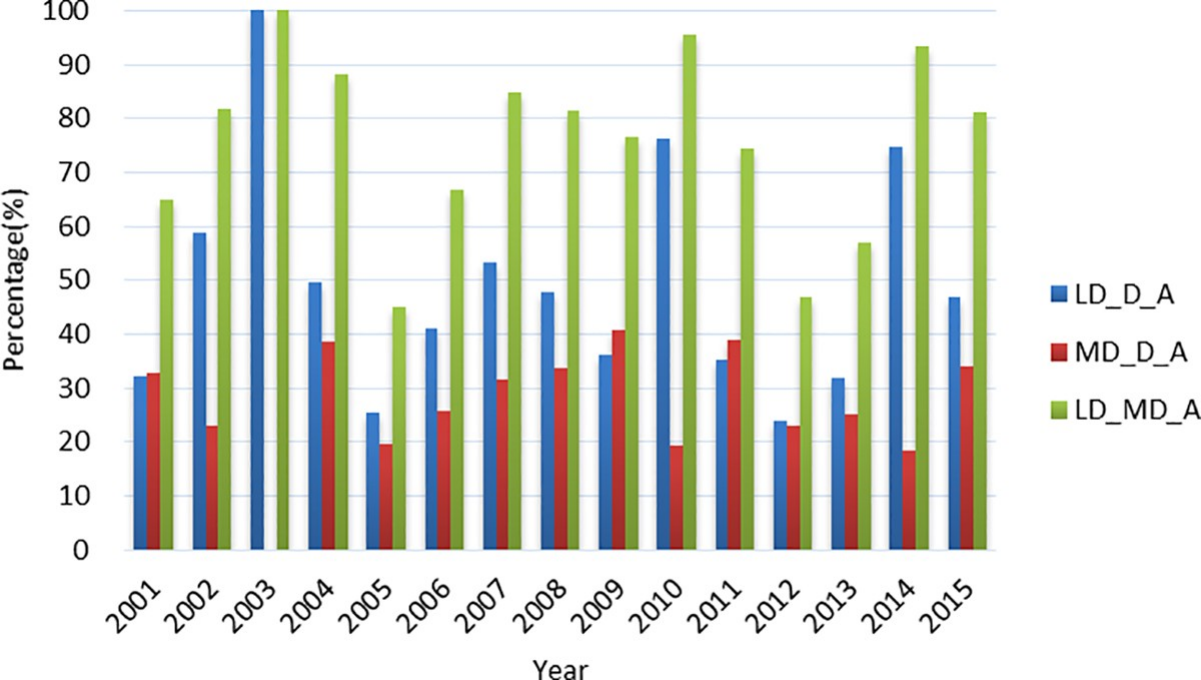
Percentage of the area of light or moderate drought to the total area of drought.

To clarify the impact of different drought severities or reductions on NPP, the drought level was divided into 5 grades (Table 1). The frequency of light drought and moderate drought were 100% and 93%, respectively. The average area percentage of light and moderate drought was 76% (Fig 4). The contribution to NPP change caused by the aggravation or alleviation of light and moderate drought was calculated.

The areas where light drought was aggravated or mitigated for two consecutive years were recorded as *A_*_*LD*_ and *R_*_*LD*_, respectively (Eqs 24 and 25); additionally, the areas where moderate drought aggravated or mitigated for two consecutive years were recorded as *A_*_*MD*_ and *R_*_*MD*_, respectively (Eqs 26 and 27). The total changes in NPP caused by the aggravation or mitigation of light drought were recorded as *S*_*↓LD_NPP*_ and *S*_*↑LD_NPP*_, respectively (Eq 28). The total changes in NPP caused by the aggravation or mitigation of moderate drought were recorded as *S*_*↓MD_NP*P_ and *S*_*↑MD_NPP*_, respectively (Eq 29), and the combined effects of light or moderate drought and LUCC causing the total changes in NPP were recorded as *S*_*↓LD_LUCC*_, *S*_*↓MD_LUCC*_, *S*_*↑LD_LUCC*_ and *S*_*↑MD_LUCC*_, respectively (Eqs 30 and 31). The total contribution of changes in NPP caused by the aggravation or mitigation of light or moderate drought were recorded as *SCR*_*↓LD_NPP*_, *SCR*_*↓MD_NPP*_, *SCR*_*↑LD_NPP*_, and *SCR*_*↑MD_NPP*_ (Eqs 32 and 33), respectively.

$$A_{\_LD}=\left\{ \begin{array}{c} 1\;(({SPEI}_{i}>\left(-0.5\right)and(-1)<{SPEI}_{i+1}\leq \left(-0.5\right))or\left((-1)<{SPEI}_{i+1}<{SPEI}_{i}\leq (-0.5)\right))\\ 0\;or\;else \end{array}\right.$$ (24)

$$R_{\_LD}=\left\{ \begin{array}{c} 1\;(\left((-1)<{SPEI}_{i}\leq (-0.5)and\;{SPEI}_{i+1}>(-0.5\right))or\left((-1)<{SPEI}_{i}<{SPEI}_{i+1}\leq (-0.5\right)))\\ 0\;or\;else \end{array}\right.$$ (25)

$$A_{\_MD}=\left\{ \begin{array}{c} 1\;(({SPEI}_{i}>(-1)and(-1.5)<{SPEI}_{i+1}\leq \left(-1\right))or\left((-1.5)<{SPEI}_{i+1}<{SPEI}_{I}\leq (-1)\right))\\ 0\;or\;else \end{array}\right.$$ (26)

$$R_{\_MD}=\left\{ \begin{array}{c} 1\;(\left((-1.5)<{SPEI}_{i}\leq \left(-1\right)and\;{SPEI}_{i+1}>(-1\right))or\left((-1.5)<{SPEI}_{i}<{SPEI}_{i+1}\leq (-1)\right))\\ 0\;or\;else \end{array}\right.$$ (27)

$$S_{↓↑(LD\_NPP)}=\left\{ \begin{array}{c} S_{↓{ED\_}_{NPP}}=\sum_{i=2004}^{2005}\left(↓({NPP}_{i+1}-{NPP}_{i})*A_{\_LD}),\;({NPP}_{i+1}<{NPP}_{i}\right)\\ S_{↑{ED\_}_{NPP}}=\sum_{i=2005}^{2006}(↑({NPP}_{i+1}-{NPP}_{i})*R_{\_LD}),\;({NPP}_{i+1}>{NPP}_{i}) \end{array}\right.$$ (28)

$$S_{↓↑(MD\_NPP)}=\left\{ \begin{array}{c} S_{↓{ED\_}_{NPP}}=\sum_{i=2004}^{2005}\left(↓({NPP}_{i+1}-{NPP}_{i})*A_{\_MD}),\;({NPP}_{i+1}<{NPP}_{i}\right)\\ S_{↑{ED\_}_{NPP}}=\sum_{i=2005}^{2006}(↑({NPP}_{i+1}-{NPP}_{i})*R_{\_MD}),\;({NPP}_{i+1}>{NPP}_{i}) \end{array}\right.$$ (29)

$$S_{↓↑(LD\_LUCC)}=\left\{ \begin{array}{c} S_{↓LD\_LCC}=\sum_{i=2004}^{2005}\left(↓({NPP}_{i+1}-{NPP}_{i})*A_{\_LD}*R_{LCC(↓)}),\;({NPP}_{i+1}<{NPP}_{i}\right)\\ S_{↑LD\_\_LCC}=\sum_{i=2005}^{2006}\left(↑({NPP}_{i+1}-{NPP}_{i})*R_{\_LD}*I_{LCC(↑)}\right),\;({NPP}_{i+1}>{NPP}_{i}) \end{array}\right.$$ (30)

$$S_{↓↑(MD\_LUCC)}=\left\{ \begin{array}{c} S_{↓LD\_LCC}=\sum_{i=2004}^{2005}\left(↓({NPP}_{i+1}-{NPP}_{i})*A_{\_MD}*R_{LCC(↓)}),\;({NPP}_{i+1}<{NPP}_{i}\right)\\ S_{↑LD\_\_LCC}=\sum_{i=2005}^{2006}\left(↑({NPP}_{i+1}-{NPP}_{i})*R_{\_MD}*I_{LCC(↑)}\right),\;({NPP}_{i+1}>{NPP}_{i}) \end{array}\right.$$ (31)

$$SCR_{↓↑(LD\_NPP)}=\left\{ \begin{array}{c} \frac{S_{↓LD\_NPP}-S_{↓LD\_LCC}}{S_{↓NPP}}*100\%,\;({NPP}_{i+1}<{NPP}_{i})\\ \frac{S_{↑LD\_NPP}-S_{↑LD\_\_LCC}}{S_{↑NPP}}*100\%,\;({NPP}_{i+1}>{NPP}_{i}) \end{array}\right.$$ (32)

$$SCR_{↓↑(MD\_NPP)}=\left\{ \begin{array}{c} \frac{S_{↓MD\_NPP}-S_{↓MD\_LCC}}{S_{↓NPP}}*100\%,\;({NPP}_{i+1}<{NPP}_{i})\\ \frac{S_{↑MD\_NPP}-S_{↑MD\_\_LCC}}{S_{↑NPP}}*100\%,\;({NPP}_{i+1}>{NPP}_{i}) \end{array}\right.$$ (33)

LD_D_A indicates the percentage of the area where light drought occurred in the area where the drought occurred (%), MD_D_A indicates the percentage of the area where the moderate drought occurred in the area where the drought occurred (%), and LD_MD_A (%) indicates the total proportion of the area with light or moderate drought to the total area where the drought occurred.

## 3. Results and analysis

### 3.1 Interannual changes in NPP and the SPEI

#### 3.1.1 Distribution and changing trends of NPP

**Fig 5:**
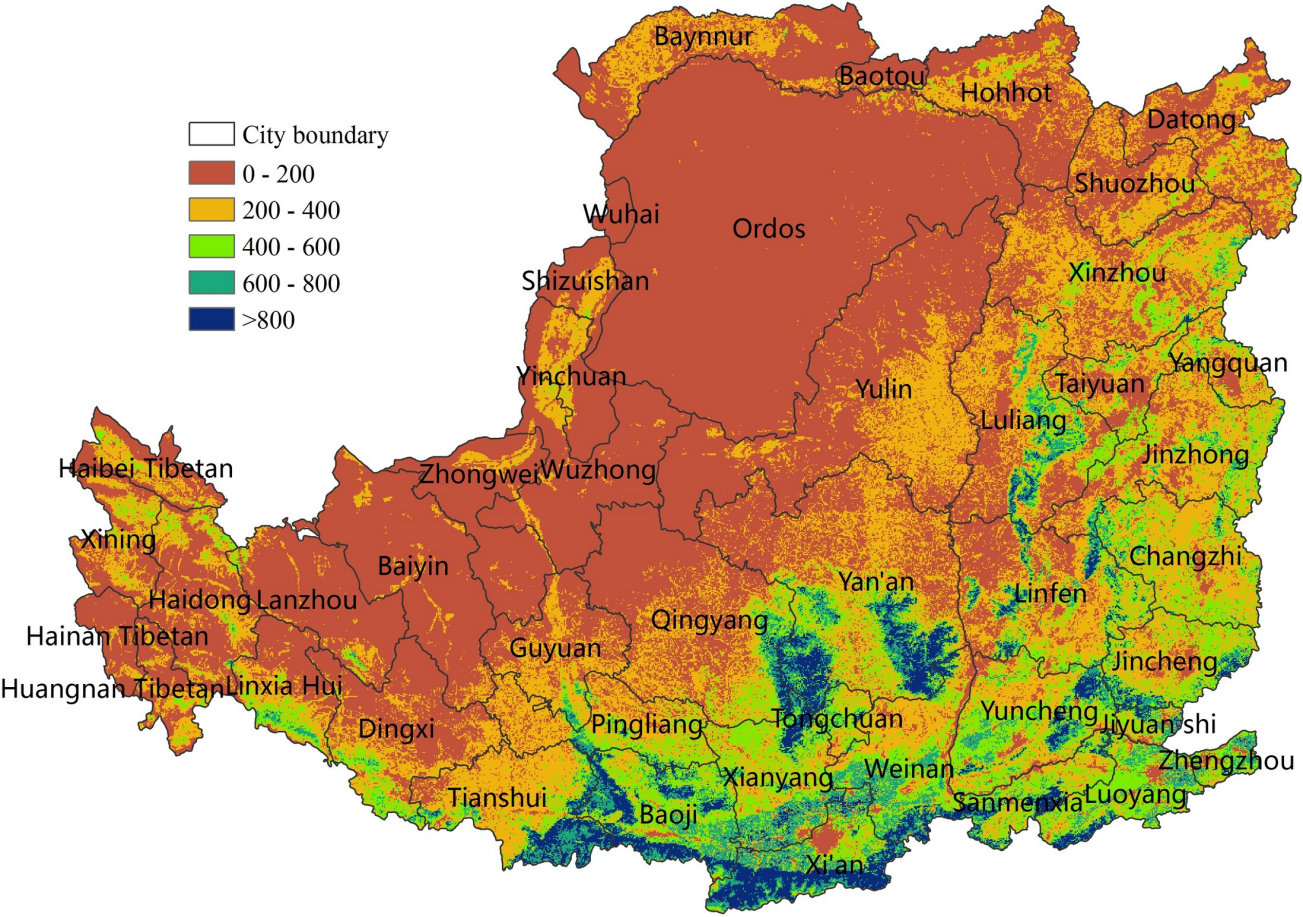
Average NPP (g C/m^2^) of the Loess Plateau from 2000 to 2015.

The average value of NPP in the Loess Plateau for 2000–2015 was generally low (Fig 5) and showed a decreasing trend from southeast to northwest. The low-value NPP region (0–200 gC/m^2^) was widely distributed and concentrated in the western gully, the north-central desert, and the northern plain. The high-value regions (600–800 gC/m^2^) were concentrated in the mountains and valley plains in the south and east. The median-value regions (200–600 gC/m^2^) were concentrated in the central and east croplands.

**Fig 6:**
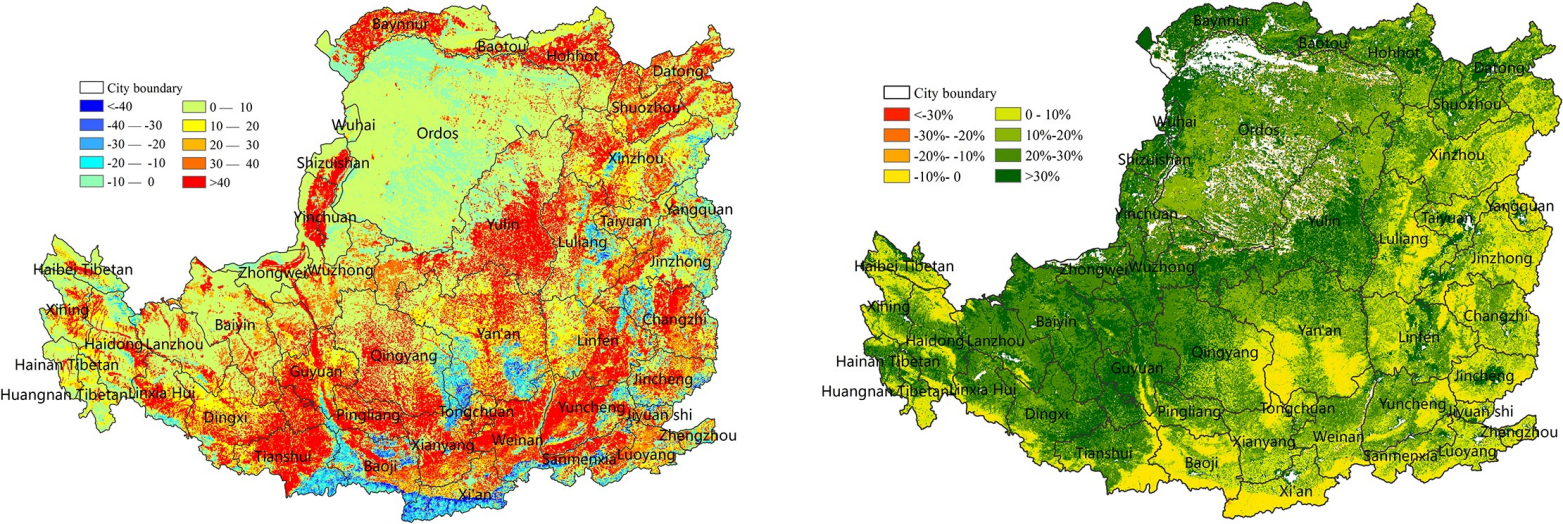
Linear change in NPP on the Loess Plateau from 2000 to 2015. (a) NPP linear change trend (gC/m^2^/ year). (b) NPP linear change rate (%).

NPP showed a linear increase trend in most regions in the Loess Plateau. The regions with fast-growth NPP were concentrated in some croplands of the western gully, the Hetao Plain of the north, and some central and southern valley plain areas, where the average annual growth of NPP was more than 40 gC/m^2^/year, and the land cover was dominant by cropland (Fig 6A). The regions with slow- growth NPP were concentrated in the Middle East and the northern edge of the Loess Plateau, where the average annual growth of NPP was lower than 20 gC/m^2^/ year, and the land cover was dominant by grassland. The sporadic distribution of forestland in the south-central region had the highest average NPP value for many years and showed a downward trend over the past 15 years, with an average annual decrease of <30 gC/m^2^/ year. The annual average NPP in some forestland decreased by >40 gC/m^2^/ year. NPP changed significantly in the western, central northern, and eastern plain, where the average annual increase rate was more than 30%, and the land cover was predominantly grassland and cropland (Fig 6B).

#### 3.1.2 Changes in the SPEI and correlation with NPP

**Fig 7:**
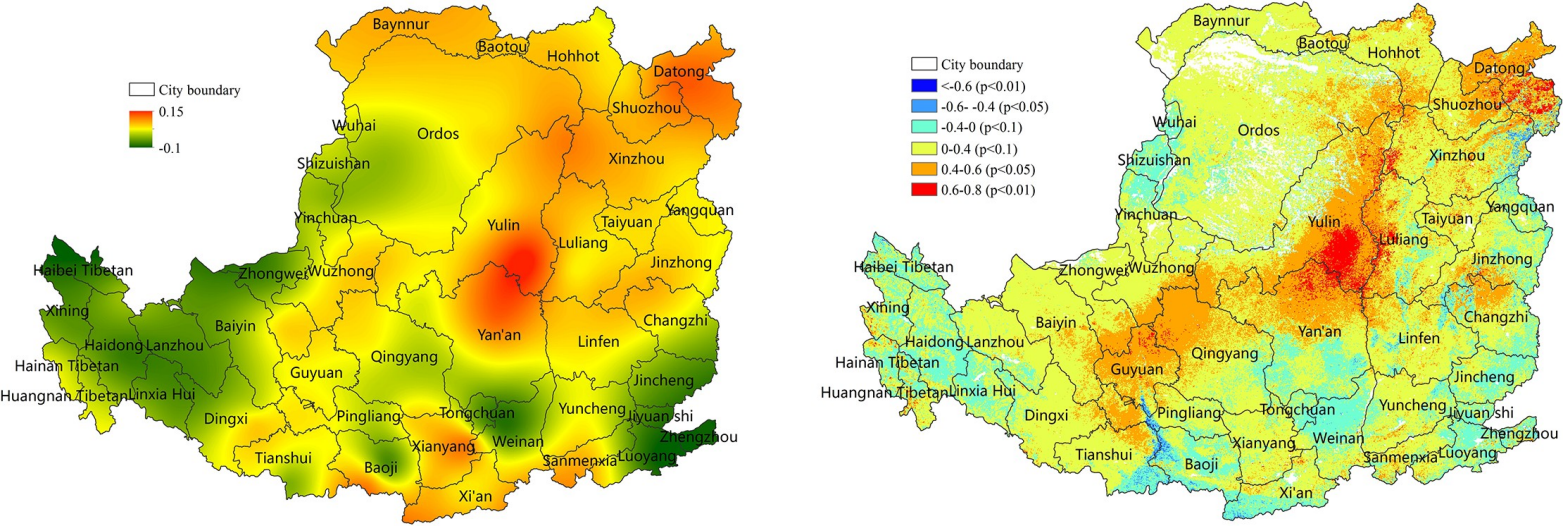
Correlation between the SPEI and NPP in the Loess Plateau from 2000 to 2015. (a) Linear trend of the SPEI. (b) Correlation coefficient between the SPEI and NPP.

The SPEI in the Loess Plateau varied from -0.1 to 0.15 over the 15 years period, and 76% of the regions showed an increasing trend (Fig 7A). The regions with fast-growth SPEI and NPP were concentrated in the north, northeast and central agricultural irrigation region (Figs 6A and 7A). Approximately 81.3% of the regions showed a positive correlation between the SPEI and NPP (Fig 7B) and showed a significant positive correlation (p<0.05) in the northeast of the Taihang Mountains, the center, and the west-central plateau gully.

Approximately 18.6% of the regions showed a negative correlation between the SPEI and NPP and were mainly concentrated in the western, south-central and eastern forestland. Human activities were the main factors that affected NPP in these regions.

### 3.2 The contribution of SPEI to NPP changes

#### 3.2.1 The contribution of hydrothermal conditions to NPP changes

**Fig 8:**
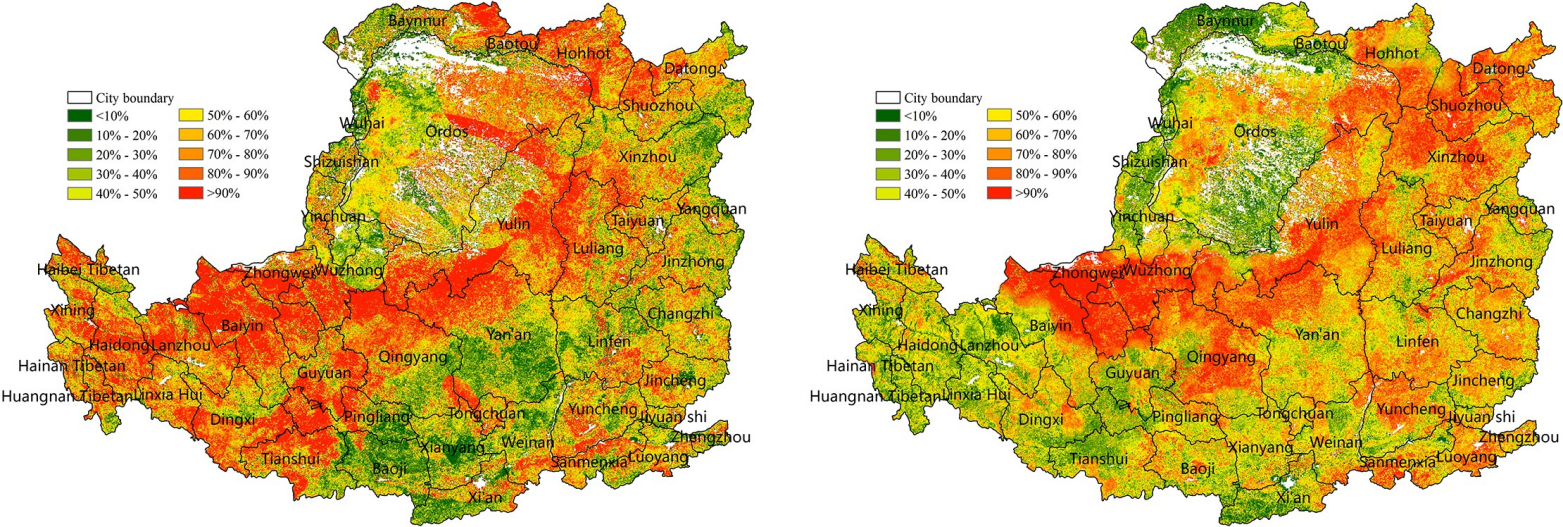
Contribution (%) of hydrothermal conditions to NPP on the Loess Plateau from 2001 to 2015 (%). (a) Contribution (%) of hydrothermal change to decreased NPP. (b) Contribution of hydrothermal condition mitigation to increased NPP (%).

The contribution of hydrothermal changes to decreased NPP was approximately 61% and exceeded 80% in the northeastern, southwestern and central regions in the Loess Plateau (Fig 8A). The land cover of these regions was grassland or cropland. The contribution of hydrothermal changes to increased NPP was approximately 59% (Fig 8B). The regions where the contribution exceeded 80% partially coincided with the high-value area of NPP reduction. Precipitation and evapotranspiration were the main factors that affected NPP change.

#### 3.2.2 The contribution of drought aggravation or mitigation to NPP changes

**Fig 9:**
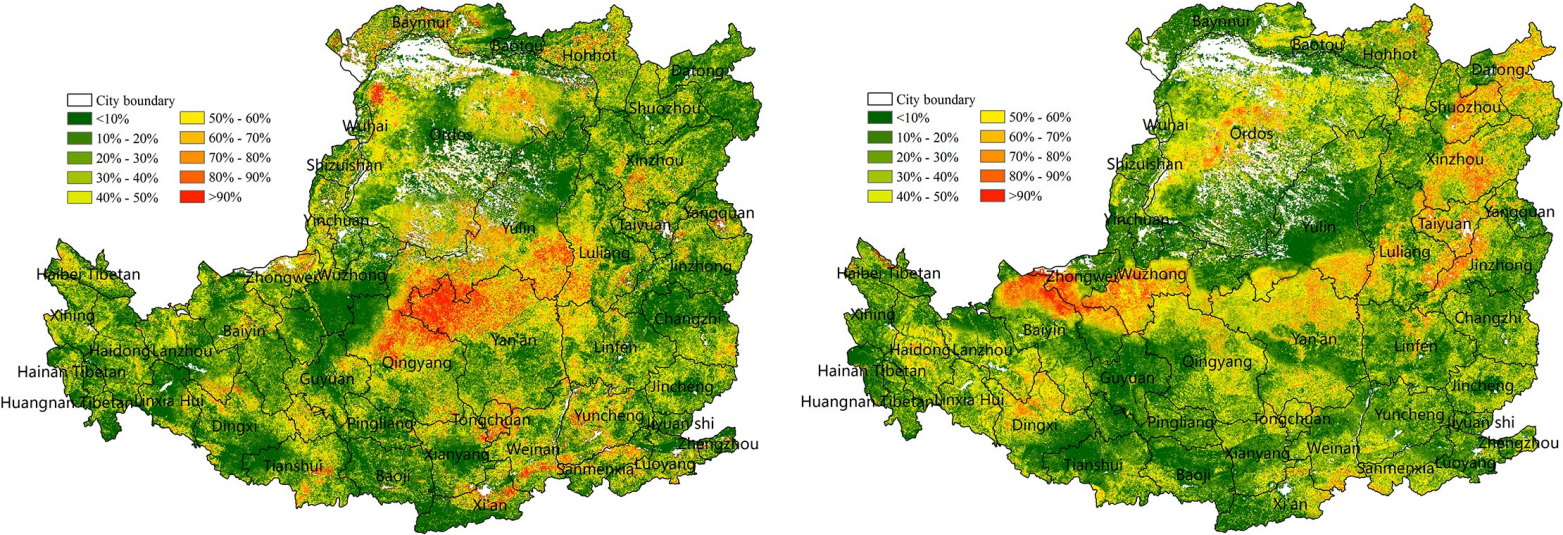
Drought contribution to NPP on the Loess Plateau from 2001 to 2015 (%). (a) Contribution of drought to NPP reduction (%). (b) Contribution of drought mitigation to NPP increase (%).

The contribution of drought or drought aggravation to decreased NPP was approximately 33% (Fig 9A). High-value areas (>40%) were evenly and widely distributed. The highest (>80%) contribution of drought to decreased NPP was observed in some grassland in the central hilly gully and plateau gully, and some cropland in the southern and central Guanzhong Plain. The bold text in Table 4 shows that the NPP decreased greatly in 8 out of 15 years, and the percentage of the drought area in 7 years was also large. Although the drought area percentage during years 13–14 years was not large, the drought developed rapidly from no drought to severe drought. These results suggested that drought was an important factor to decreased NPP.

**Table 4:** Arid area percentage and average NPP reduction.

Year	Drought area (%)	D_NPP (g/m^2^)
01_02	12	5
02_03	0	4
**03_04**	**31**	**20**
**04_05**	**60**	**21**
05_06	29	9
06_07	10	7
**07_08**	**42**	**18**
**08_09**	**49**	**17**
09_10	14	9
**10_11**	**29**	**44**
11_12	15	7
**12_13**	**30**	**24**
**13_14**	8	**31**
**14_15**	**35**	**24**

Year indicates two consecutive years, e.g., 01_02 indicates 2001–2002; drought area indicates the drought area percentage; and D_NPP indicates the average reduction in NPP between two consecutive years.

The contribution of drought mitigation to increased NPP was approximately 32% and greater than 40% in most areas (Fig 9B). Particularly, the contribution of the hilly gully in the northeast and gully in the western plateau was more than 80%, where the land cover was predominantly grassland, which was sensitive to drought mitigation. In addition, the high contribution areas (>50%) in the central and eastern regions and the fast-growth areas of NPP for 2000–2015 were highly consistent. Drought alleviation was an important factor that increased NPP.

#### 3.2.3 The contribution of different levels of drought to NPP changes

**Fig 10:**
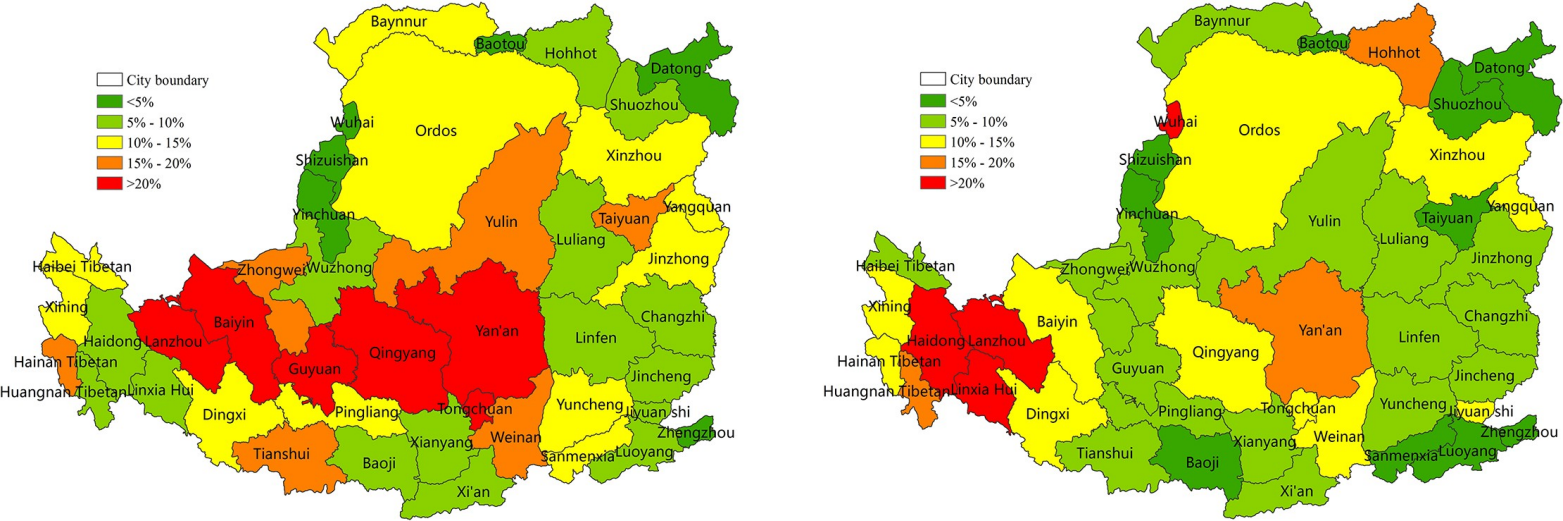
The contribution of NPP reduction caused by drought aggravation in 2001–2015 at the prefecture level on the Loess Plateau. (a) The contribution of NPP reduction caused by the drought changes from no drought to light drought and the degree of light drought increased within the same drought level. (b) The contribution of NPP reduction caused by the drought changing from no drought or light drought to moderate drought, and the degree of moderate drought increased within the same drought level.

**Fig 11:**
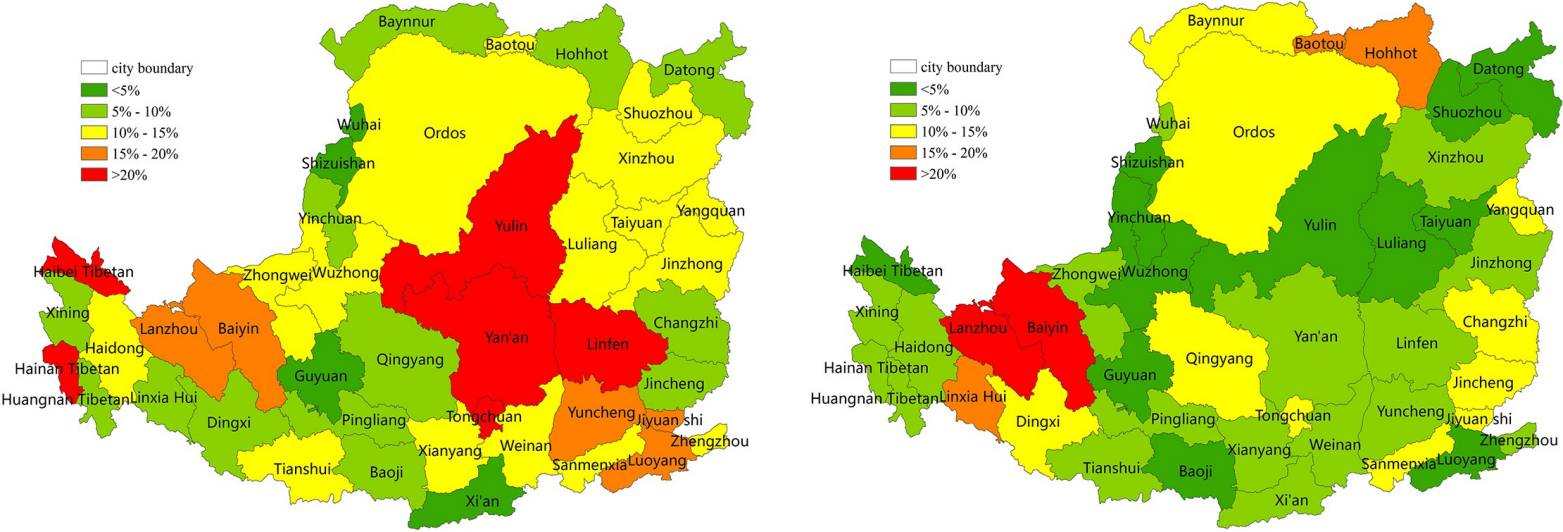
The contribution to NPP increase caused by drought relief in 2001–2015 at the prefecture level on the Loess Plateau. (a) The contribution to NPP increase caused by drought relief from light drought to no drought, and the degree that light drought was alleviated within the same drought level. (b) The contribution to NPP increase caused by drought relief from moderate drought to light drought or no drought, and the degree of moderate drought that was alleviated within the same drought level.

To help local governments maintain regional ecosystem stability and security, the contribution of light and moderate drought aggravation or mitigation to the decrease or increase in NPP at the prefecture and city level was quantified (Figs 10 and 11). The contribution of light drought to NPP was larger than moderate drought. According to Fig 10A, the contribution of light drought aggravation to decreased NPP was 13% and included the situation of drought from no drought to light drought and exacerbations in the same drought level. High values (>20%) were distributed in the gully area of the mid-west. In addition, 6 of the 44 cities had a contribution of >20%, and Yan'an was the highest (23.1%). Similarly, the contribution of moderate drought aggravation to decreased NPP was 10%, and included the situation of drought from no drought or light drought to moderate drought and exacerbations in the same drought level. The high-value area (>20%) was distributed in the western plateau gully and the central edge of the agricultural irrigation. In addition, 4 of the 44 cities had a contribution of >20%, and Linxia was the highest (28%).

The contribution of the increase in NPP caused by the alleviation of light or moderate drought is shown in Fig 11. The alleviation of light drought contributed to approximately 12.7% of the NPP increase. The high-value area (>20%) was distributed in the western marginal gully and the central gully or plain. In addition, 6 of the 44 cities had a contribution of >20%, and Hainan Tibetan was the highest (41%) (Fig 11A). The alleviation of moderate drought contributed to approximately 9.4% of the NPP increase. High-value areas (>20%) were distributed in the gully area of the western plateau, and the land cover was mostly grassland. In addition, 2 of the 44 cities had a contribution of >20%, and Baiyin was the highest (32%) (Fig 11B).

### 3.3 The contribution of LUCC to NPP changes

#### 3.3.1 The contribution of land cover change to NPP changes

**Fig 12:**
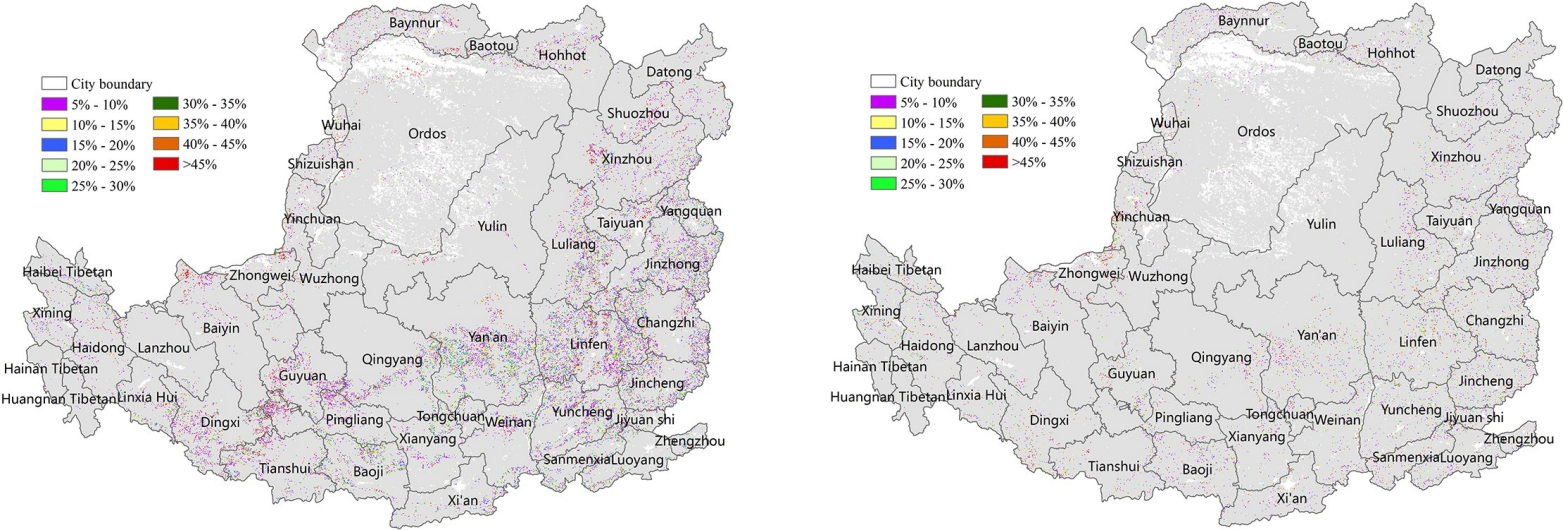
*The contribution of land cover change to NPP on the Loess Plateau from* 2001 to 2015 (%). (a) The contribution of land cover change to decreased NPP (%). (b) Contribution of land cover change to increased NPP (%).

The contribution of land cover change to the decrease or increase in NPP was generally smaller than that of drought, and the spatial distribution was uneven (Fig 12A and 12B). The contribution of land cover changes to decreased NPP was generally between 5% and 10%, with an average of approximately 1.4%. The regions with decreased NPP caused by land cover change were concentrated in the south-central gully and the eastern Taihang Mountain, where the land cover was predominantly forestland, which is consistent with the NPP reduction region shown in Fig 6. The conversion of land cover was mainly from forestland to non-forestry land. The contribution of some forestland in Ziwuling was between 25% and 30%, and greater than 60% in Luliang Mountain. The areas where land cover change contributed to increased NPP were concentrated in the cropland or grassland around the south-central and eastern forest, with an average contribution of approximately 0.5%. The conversion of land cover was mainly from grassland or cropland to cropland or forestland. The contribution of some grassland areas on the northern border of Zhongwei and Wuzhong was higher than 50%.

#### 3.3.2 The contribution of major land-use change to NPP changes

**Fig 13:**
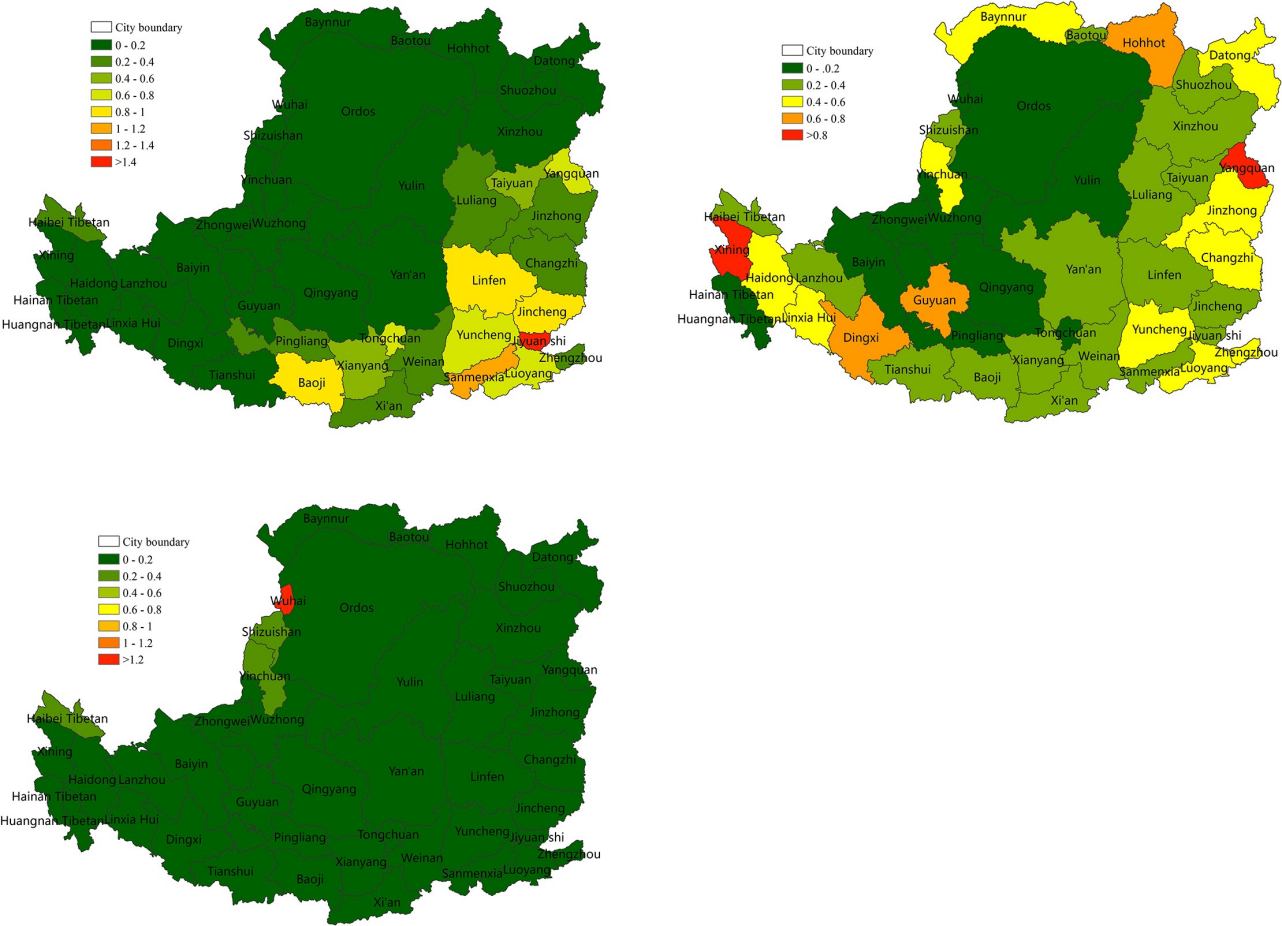
The contribution of land-use change to decreased NPP on the Loess Plateau in 2000–2015 (%). (a) The contribution of conversion of forestland to non-forestry land to decreased NPP (%). (b) The contribution of conversion of cropland to grassland or non-NPP main production land to decreased NPP (%). (c) The contribution of conversion of grassland to non-NPP main production land to decreased NPP (%).

To help the local governments establish a healthy ecosystem, we focused on quantifying the contribution of the main land uses (forestland, cropland and grassland) to each other or to non-NPP major production land uses (urban land, water, and unused land) to NPP on the prefecture and city level. The average contribution of forestland, cropland and grassland conversion to decreased NPP in each urban is shown in Fig 13A and 13C, respectively. Compared with SPEI, the average contribution of the main land uses to NPP was relatively low, i.e., below 5%. The average contribution of conversion from forestland to non-forestry land to decreased NPP was approximately 0.2% (Fig 13A). The high-value regions were concentrated in the Taihang Mountain and valley plain in the southeast and south-central area. In addition, 5 of the 44 cities had a contribution of >0.8%, and Jiyuan was the highest (1.6%). The average contribution of conversion from cropland to grassland or non-NPP main production land to decreased NPP was approximately 0.3% (Fig 13B). The high-value region were concentrated in the northern Hetao Plain, the western plateau gully and the eastern Fenhe Valley, and Yangquan was the highest (0.84%). The average contribution of conversion from grassland to non-NPP main production land to decreased NPP was approximately 0.04%, and Wuhai was the highest (1.3%) (Fig 13C).

**Fig 14:**
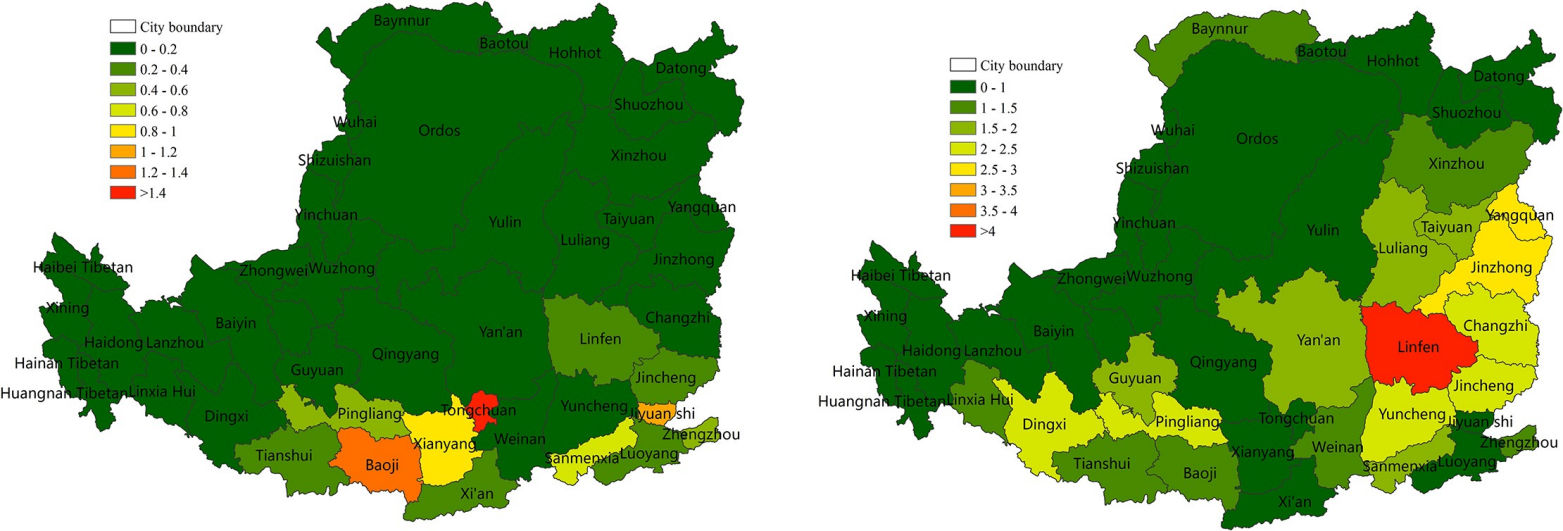
The contribution of land-use change to increased NPP on the Loess Plateau from 2001 to 2015 (%). (a) The contribution of conversion of cropland to forestland to increased NPP (%). (b) The contribution of grassland conversion to cropland or forestland to increased NPP (%).

The average contribution of cropland and grassland conversion to increased NPP in each urban area is shown in Fig 14A and 14B, respectively. The contribution was generally low, i.e., less than 5%. The average contribution of the conversion of cropland to forestland to increased NPP was approximately 0.1%. The high-value areas were concentrated in the plain south of the central region. In addition, 3 of the 44 cities had a contribution of >0.8%, and Tongchuan was the highest (1.4%) (Fig 14A). The average contribution of conversion of grassland to cropland or forestland to increased NPP was approximately 1.1%. In addition, 20 of the 44 cities had a contribution >0.8%, and Linfen was the highest (4%) (Fig 14B). Among the three land uses, the conversion of cropland to grassland or non-NPP main production land had the highest impact on the reduction of NPP, and the conversion of grassland to cropland or forestland had the highest impact on increased NPP.

## 4. Discussion

### 4.1 Differentiating the effects of hydrothermal, drought, and land use and land cover change (LUCC) on NPP variations

According to our study, the relationships between NPP and SPEI and between NPP and LUCC quantitatively represented the responses of NPP to hydrothermal conditions, drought and LUCC. We found that hydrothermal conditions had the strongest impact on NPP, followed by drought. The influence of hydrothermal conditions and drought on NPP mainly showed a "planar" distribution in space, i.e., these two factors mainly affected the change of NPP in a large area. In contrast to SPEI, the LUCC changed only slightly in the entire Loess Plateau and mainly occurred in small local areas. The influence of LUCC on NPP mainly shows a "point" distribution in space, i.e., LUCC had a relatively small impact on NPP in the entire Chinese Loess Plateau and a relatively large impact on local NPP. The contribution of LUCC to NPP changes in some local areas was greater than 80% (Fig 12A and Fig 12B). These results suggest that LUCC is the controlling factor of NPP change in some local areas of the Loess Plateau, but has little influence on the entire Loess Plateau.

### 4.2 Measuring the contribution of major land-use change to NPP variations reflects the restoration effect of the ecological environment

**Fig 15:**
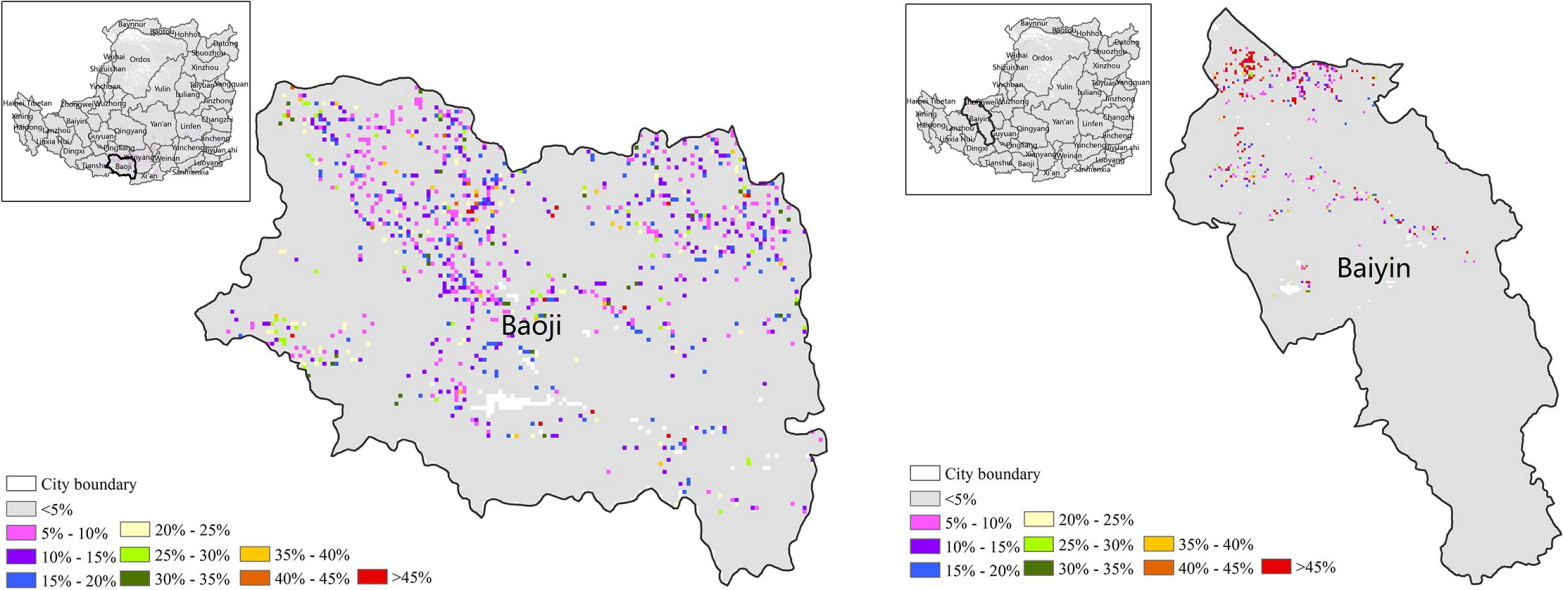
The contribution of conversion from cropland to forestland to increased NPP. (a) The contribution of conversion from cropland to forestland to increased NPP. (b) The contribution of conversion from unused land to grassland, forestland and cropland to increased NPP.

Owing to the fragile eco-environment, terrain fragmentation, serious soil erosion and the long term negative effects of human activity, the eco-environment in the Loess Plateau was degraded. Through the implementation of ecological protection measures, including China's Grain to Green Project (GTGP) and Land Desertification Control Project (LDCP) beginning in 1990, the ecological environment has been restored to a certain extent [30]. NPP can assessed the production capacity and sustainable development of eco-environment. The restoration effect of the ecological environment for 2000–2015 in the Loess Plateau can be quantitatively described through the contribution of major land-use change to the increase in NPP. The results showed that the conversion of cropland to forestland was the main factor for increased NPP in some areas. Taking Baoji as example, the contribution of the cropland to the increase in NPP was quantified, and showed that most cropland was converted to forest, with a contribution greater than 45% in the south (Fig 15A). The forest coverage of Baoji increased from 45% to 51% (675 km^2^), the cropland coverage decreased from 47% to 44% (278 km^2^), and the NPP increased by 11 gC/m^2^/year during the 15-year period. These results are consistent with the previous research by Xu Yuxia for the return of cropland to forest for 1999–2015 in Baoji [31].

Similarly, the contribution of wasteland and sandy land converted to forestland to the increase in NPP was generally greater than 45% in Baiyin (Fig 15B). The wasteland and sandy land in Baiyin was reduced by 407 km^2^ and the coverage rate decreased from 5% to 3%; the forestland increased by 268 km^2^, and the coverage rate increased from 1% to 3%. In addition, NPP has increased by 14 gC/m^2^/ year.

The results suggest that the GTGP and LDCP effectively promoted increased NPP and eco-environmental restoration. The implementation of these measures can promote eco-environmental safety and stability.

### 4.3 The accuracy of NPP from its estimation model

The major error of NPP estimates is the accuracy of the NPP product algorithm. This accuracy is associated with the parameter selection and construction methods of different models. The light energy utilization model we adopted based on the GLM_PEM is a productivity model that is mainly driven by remote sensing data. It can make full use of the advantages of remote sensing to produce high-resolution estimates of regional-scale vegetation, especially for forestland NPP [23] Liang et al. found that the correlation coefficient between the observations of gross primary production (GPP) from flux tower and NPP calculated by the GLM_PEM model was higher than that by the Carnegie-Ames-Stanford approach (CASA) model, which indicated that the accuracy of GLM_PEM was better than that of CASA [32]. The GLM_PEM model has been successfully applied to estimate NPP and GPP in global terrestrial ecosystems [33–36]. Based on these validations, we believe that the GLM_PEM model is suitable for analysis in this study.

### 4.4 Advantages and limitations of the research methods

Drought and LUCC are the main factors to affect NPP. [37–39]. The impact of drought and LUCC to NPP were quantified and the mechanisms underlying the effect of drought and LUCC on NPP were clarified. These results provide an important reference value for future research on the carbon cycle and regional ecological environmental restoration. It is difficult to distinguish the dominant factors of NPP change in the overlapping region of drought and LUCC. In this study, overlapping areas generally show the influence of LUCC on NPP, while the contribution of drought to NPP is underestimated. This measurement method limitation should be optimized in the future.

## 5. Conclusions

We analyzed the temporal and spatial characteristics of NPP in the Chinese Loess Plateau during 2000–2015 and quantified the contributions of hydrothermal conditions, drought and LUCC. In particular, we quantified the major land uses and different degrees of drought aggravation or mitigation to changes in NPP. The main findings are summarized as follows:

(1) The15-years average NPP was approximately 227 gC/m^2^ and decreased from southeast to northwest. A linear increasing trend of NPP was observed in cropland and grassland, with the largest increasing value in cropland and the fastest growth rate in grassland, and a linear decreasing trend was observed in forestland.

(2) Approximately half of the NPP change was attributed to hydrothermal conditions and drought. Approximately 61% of the NPP decrease and approximately 59% of the NPP increase were affected by hydrothermal conditions, respectively, while approximately 33% of the NPP decrease and approximately 32% of the NPP increase were affected by drought, respectively. Among different levels of drought, light drought contributes the most to the NPP change.

(3) For the entire loess plateau, LUCC has little effect on the increase and decrease of NPP, but for some local regions, the change contribution of LUCC to NPP reaches half (45%). The contribution of major land uses to the NPP change indicates that GTGP and LDCP effectively promoted increased NPP and eco-environment restoration.

## Supporting information

S1 File (DOCX)

S1 Data (DOCX)
