# Supplementary material for: Quantifying the impacts of drought and ecological restoration on net primary production changes in the Chinese Loess Plateau
Source: PLoS One. 2020 Sep 24;15(9):e0238997. doi: 10.1371/journal.pone.0238997 (PMC7514050; doi:10.1371/journal.pone.0238997)
Supplement: S1 File — (DOCX) [file pone.0238997.s001.docx]

Quantifying the impacts of drought and ecological restoration on net primary production changes on the Chinese Loess Plateau

Xiaowei Jiang^1¶^, Jiangjun Bai^1¶^

^1^ School of Geography and Tourism, Shaanxi Normal University, Xi’an, China

***** Correspondence author

E-mail: 648762060@qq.com (XJ)

¶These authors contributed equally to this work.

# Abstract:

Net primary production (NPP) can regulate global climate change and the carbon balance. Although scholars have qualitatively studied the influencing factors of NPP, few scholars have quantified the contribution of different degrees of drought aggravation or mitigation and major land use changes to NPP changes. Based on an analysis of the temporal and spatial characteristics of NPP on the Loess Plateau from 2000 to 2015, we quantified the contribution of drought, land cover change, and hydrothermal conditions to changes in NPP. Particularly, we analyzed the contribution of major land use change and different drought levels on NPP. Our research showed that the average NPP over 15 years was generally low, with an average of approximately 227 gC/m^2^, and it decreased from southeast to northwest. NPP showed a linear increasing trend over 15 years, and forest land showed a linear decreasing trend. The data showed that changes in hydrothermal conditions had the strongest impact on NPP (~61%), followed by drought (~33%), and land cover change had the weakest impact (~1.4%). In particular, ~13% of the NPP reduction was affected by the aggravation of drought to light drought, ~10% was affected by the aggravation of drought to medium drought, and ~0.3% was affected by the conversion of cropland to grassland or non-NPP main production land. Moreover, ~12.7% of the NPP increase was affected by the alleviation of light drought, ~9.4% was affected by the alleviation of moderate drought, and ~1.1% was affected by the conversion of grassland to cropland or forest land. The results were relatively accurate and the mechanisms underlying the effect of drought and land use change on NPP were clarified, thus providing insights into the development of healthy ecosystems.

**Keywords:** net primary production, drought, land cover change, MODIS, hierarchical quantification, ecological restoration, the Loess Plateau.

# Introduction

Since the industrial revolution, the massive burning of fossil fuels has led to the increasing release of greenhouse gases, such as CO_2_ [[1](#_ENREF_1), [2](#_ENREF_2)]. Subsequently, a series of environmental problems have occurred. As the first step of carbon sequestration in an ecosystem [[3](#_ENREF_3)], net primary production (NPP) represents the main method of mitigating the increasing concentration of greenhouse gases. NPP plays an important role in regulating global climate change and the carbon balance, and it is also an important indicator for evaluating the sustainable development of terrestrial ecology [[4](#_ENREF_4)]. Research on the relevant influencing factors and driving mechanisms of NPP is particularly important. The academic community generally believes that climate factors and land cover change (LCC) are the main factors that influence NPP [[5-7](#_ENREF_5)]. Cleveland et al. found that climate factors are the strongest predictors of NPP changes in tropical rain forests [[6](#_ENREF_6)]. Dailiang et al. found that land use change is one of the main factors affecting NPP change [[7](#_ENREF_7)]. The main factors underlying drought, such as precipitation, temperature and evapotranspiration, restrict the growth of vegetation and become the important factors for NPP [[8](#_ENREF_8)]. Zhao et al. found that large-scale drought led to a decrease in global NPP [[9](#_ENREF_9), [10](#_ENREF_10)]. In general, scholars have quantitatively studied the factors that influence NPP. However, accurately evaluating the specific contributions of each influencing factor to changes in NPP is difficult. Furthermore, few scholars have quantified the effect of different degrees of drought aggravation or mitigation and major land use change to NPP change [[11-13](#_ENREF_11)].

The Loess Plateau of China is located in the semi-arid and semi-humid zone, and it is one of the most vulnerable ecological environments in the world and shows sensitivity to climate change and human activities [[14](#_ENREF_14)]. In recent years, the frequency of drought events has affected the change in NPP. Studies have found that drought has generally shown a trend of alleviation [[15](#_ENREF_15)], which has a profound impact on changes in NPP. Due to the implementation of a series of ecological protection measures, such as China's Grain to Green Project (GTGP), the ecological environment in the region has been restored, which also affects the changes in NPP [[16](#_ENREF_16)]. To promote the sustainable development of the region, we should understand the temporal and spatial changes of NPP and the role and contribution of drought and LCC in the change process. Based on an analysis of the temporal and spatial characteristics of NPP on the Loess Plateau from 2000 to 2015, we quantified the contribution of hydrothermal conditions, drought, and LCC to the change in NPP. Particularly, we analyzed the influence of major land use change on NPP as well as the influence of drought severity or mitigation on NPP. The research results are accurate and provide an important reference value for future research on the carbon cycle, regional ecological environment balance, and regional ecological environment restoration.

# Data and methodology

## 2.1 Study Area

The Loess Plateau is located in the middle of the Yellow River at 100° 54′~114° 33′E, 33° 43′~41° 16′N, and it spans 7 provinces and 44 prefecture-level cities, with a total area of 624,000 km^2^ (Fig 1). It can be divided into three parts: east, middle, and west, with Liupan Mountain and Luliang Mountain serving as the boundaries. The landforms of this region can be divide into plateau gullies, hilly gullies, mountains, loess gullies and river valley plains. The average annual temperature is 3.6~14.3°C, and the water resources are scarce [[17](#_ENREF_17)]. The average annual precipitation is 150-750 mm, which is concentrated from July-September mostly in the form of rainstorms. Evaporation is generally higher than precipitation. The MODIS land cover product (MCD12Q1) in this area shows that there are 13 land cover types, which are mainly grassland and cropland, followed by forest land. Grassland accounts for 61% of the total area, cropland accounts for 23% and forest land accounts for 10% (using 2011 as an example) (Fig 2).

**Fig 1. Provincial and prefecture-level administrative maps of the Loess Plateau.**

**Fig 2. Land cover distribution derived from MCD12Q1 in 2010 for the Loess Plateau.** Land cover types include evergreen needleleaf forest (ENF), deciduous broadleaf forest (DBF), mixed forest (MF), closed shrublands (CSH), open shrublands (OSH), woody savannas (WSA), grasslands (GRA), permanent wetlands (WET), croplands (CRO), urban and built-up (URB), cropland/natural vegetation mosaic (CNV), barren or sparsely vegetated (BSV), and water (WAT).

## 2.2 Data collection and pre-processing

The NPP data and the Loess Plateau boundary vector diagram are from the Resource and Environmental Data Cloud Platform of the Chinese Academy of Sciences (<http://www.resdc.cn/Default.aspx>). Its projection coordinate system is Alberts_1940. The NPP data came from a national dataset based on the calculation of the light energy utilization model GLM_PEM [[18](#_ENREF_18)]. The spatial resolution was 1 km.

Precipitation and temperature are the main climatic factors affecting NPP changes in arid and semiarid regions [[19-21](#_ENREF_19)]. The standardized precipitation evaporation index (SPEI) is a drought monitoring index that comprehensively considers precipitation and evapotranspiration [[22](#_ENREF_22)]. Compared with the standard precipitation index (SPI), the SPEI can reflect the impact of temperature on drought; and compared with the Palmer drought severity index (PDSI), the SPEI has multiple time scale advantages [[23](#_ENREF_23)]. In this study, the SPEI came from the global monthly SPEI dataset, which had a time scale of 12 months, and its spatial resolution is 0.5° (http://digital.csic.es/handle/10261/128892/). Then, we resampled the data to 1 km and projected the data via the Alberts_1940 projection to analyze the impact of drought on NPP. The required meteorological data for the SPEI are from the TRMM.

Land cover/land use is major factor affecting NPP [[24](#_ENREF_24), [25](#_ENREF_25)]. We used MODIS land cover (MCD12Q1, 500 m) data products from 2001 to 2015 (https://ladsweb.modaps.eosdis.nasa.gov/search/), and we resampled the data to 1 km and projected them via Alberts_1940. The ground cover classification system was provided by the International Geosphere-Biosphere Program (IGBP) land cover classification scheme (Fig 2).

Since MODIS land cover products for 2001-2015 are available, this study calculates the contribution of drought and LCC to NPP in the Loess Plateau from 2001 to 2015.

## 2.3 Analysis of temporal changes in NPP and SPEI

We analyzed the linear trend of NPP and the SPEI in the study area from 2000 to 2015. The calculation formulas of the linear change value *α* and the linear change rate *β* are shown in

Eqs 1 and 2, respectively. We calculated the correlation coefficient R of NPP and the SPEI-12 (Eq 3).

$\alpha=\frac{\sum_{i=1}^{n} t_{i}x_{i}-\frac{1}{n}\sum_{i=1}^{n} t_{i}\sum_{i=1}^{n} x_{i}}{\sum_{i=1}^{n} {t_{i}}^{2}-\frac{1}{n}\left（ \sum_{i=1}^{n} t_{i} \right）^{2}}$ (1)

*β*=*α*/$\bar{x}$*16*100% (2)

*R*=$\frac{\sum_{i=1}^{n} (NPP-\bar{NPP})(SPEI-\bar{SPEI})}{\sqrt{\sum_{i=1}^{n} {(NPP-\bar{NPP})}^{2}\sum_{i=1}^{n} {(SPEI-\bar{SPEI})}^{2}}}$ (3)

In the formulas, *α* is the value of linear tendency; n is the number of years in the monitoring period, *n*=16; i is the serial number of the year, *i*=1,2,3… ..16; *t_i_* is the *i*-th year; x_i_ is the NPP value or SPEI value in year *i*; *β* is the linear change rate; $\bar{x}$ is the multiyear average value of NPP or SPEI; $(\bar{NPP)}$ is the multiyear average value of NPP; and $(\bar{SPEI)}$ is the multiyear average value of the SPEI.

## 2.4 Quantifying the contribution of drought, land cover change, and hydrothermal conditions to changes in NPP

According to the international standard for classifying drought grades based on the SPEI, we classified droughts as drought-free, light drought, moderate drought, severe drought and extreme drought (Table 1).

**Table 1. Classification of drought grades based on the SPEI**

| **Drought grade** | **SPEI value** |
| --- | --- |
| drought-free | SPEI＞-0.5 |
| light drought | -1＜SPEI≤-0.5 |
| moderate drought | -1.5＜SPEI≤-1 |
| severe drought | -2＜SPEI≤-1.5 |
| extreme drought | SPEI≤-2 |

We defined SPEI>-0.5 as normal or humid and SPEI≤-0.5 as drought or severe drought. For two consecutive years, the area with drought or aggravated drought was recorded as D (Eq 4) and the normal or wet area was recorded as N (Eq 5). The SPEI can reflect the change in hydrothermal conditions, and its increase or decrease is denoted as the SPEI (d) (Eq 6) and the SPEI (I) (Eq 7), respectively. In Eqs 4-7, i represents the year.

*D*=$\left\{ \begin{aligned} 1 \left( \left( \left. {SPEI}_{i}>\left( \left. -0.5 \right) \right.\mathrm{and}{SPEI}_{i+1}\leq\left( \left. -0.5 \right) \right. \right) \right.\mathrm{or}\left( \left. {SPEI}_{i+1}<{SPEI}_{I}\leq\left( -0.5 \right) \right) \right. \right) \\ 0 or else \end{aligned} \right.$ (4)

*N*=$\left\{ \begin{aligned} 1 \left( \left( \left. {SPEI}_{i}\leq\left( \left. -0.5 \right) \right.\mathrm{and} {SPEI}_{i+1}>\left( \left. -0.5 \right) \right. \right) \right.\mathrm{or}\left( \left. {SPEI}_{i}<{SPEI}_{i+1}\leq\left( -0.5 \right) \right) \right. \right) \\ 0 or else \end{aligned} \right.$ (5)

*SPEI (d)*=$\left\{ \begin{aligned} {1 SPEI}_{i+1}<{SPEI}_{i} \\ 0 or else \end{aligned} \right.$ (6)

*SPEI (I)*=$\left\{ \begin{aligned} {1 SPEI}_{i+1}>{SPEI}_{i} \\ 0 or else \end{aligned} \right.$ (7)

We divided the study area into 1 km × 1 km grids. Considering the ability of different land uses to generate NPP (Fig 3) and based on previous research [[26](#_ENREF_26), [27](#_ENREF_27)], we determined that LCC causes NPP to decrease or increase (Table 2). *R__LCC(↓_*_)_ and *I__LCC(↑)_* indicate the area where LCC caused a decrease or increase in NPP, respectively (Eq 8 and 9).

**Fig 3. Average NPP (gC/m^2^) from different land use types on the Loess Plateau from 2000 to 2015.**

*R__LCC(↓_*_)_=$\left\{ \begin{aligned} 1\left\{ \begin{aligned} \mathrm{LCC}\left( \left. DBF,MF \right) \right.to LCC\left( \left. CSH,CNV,WSA,CRO,GRA \right) \right. \\ or LCC\left( \left. CSH,CNV,WSA,ENF,CRO \right) \right.to LCC\left( \left. GRA,WET,URB \right) \right. \\ \mathrm{orLCC}\left( \left. GRA,WET \right) \right.to LCC\left( \left. WAT,OSH \right) \right. \end{aligned} \right. \\ 0 or else \end{aligned} \right.$ (8)

*I__LCC(↑)_*=$\left\{ \begin{aligned} 1\left\{ \begin{aligned} \mathrm{LCC}\left( \left. WAT,OSH \right) \right.to LCC\left( \left. GRA,WET \right) \right. \\ or LCC\left( \left. GRA,WET \right) \right.to LCC\left( \left. CSH,CNV,WSA,MF,ENF,CRO \right) \right. \\ \mathrm{orLCC}\left( \left. GRA,CRO,WSA,CSH \right) \right.to LCC\left( \left. DBF,MF \right) \right. \end{aligned} \right. \\ 0 or else \end{aligned} \right.$ (9)

**Table 2. Changes in NPP caused by land cover type conversion**

|  | **DBF** | **MF** | **CSH** | **CNV** | **WSA** | **ENF** | **CRO** | **GRA** | **WET** | **URB** | **OSH** | **WAT** |
| --- | --- | --- | --- | --- | --- | --- | --- | --- | --- | --- | --- | --- |
| **DBF** | ○ | × | ↓ | ↓ | ↓ | × | ↓ | ↓ | × | × | × | × |
| **MF** | × | ○ | ↓ | ↓ | ↓ | × | ↓ | ↓ | × | × | × | × |
| **CSH** | ↑ | ↑ | ○ | ○ | ○ | ○ | ○ | ↓ | × | × | × | × |
| **CNV** | × | × | ○ | ○ | ○ | × | ○ | ↓ | × | ↓ | × | × |
| **WSA** | ↑ | ↑ | ○ | ○ | ○ | ○ | ○ | ↓ | ↓ | ↓ | × | × |
| **ENF** | × | × | ○ | ○ | ○ | ○ | ○ | ↓ | × | × | × | × |
| **CRO** | ↑ | ↑ | ○ | ○ | ○ | ○ | ○ | ↓ | ↓ | ↓ | × | × |
| **GRA** | ↑ | ↑ | ↑ | ↑ | ↑ | ↑ | ↑ | ○ | ○ | ○ | ↓ | ↓ |
| **WET** | × | ↑ | × | ↑ | ↑ | ↑ | ↑ | ○ | ○ | × | × | ↓ |
| **URB** | × | × | × | × | × | ↑ | × | × | × | ○ | × | × |
| **OSH** | × | × | × | × | × | × | × | ↑ | × | × | ○ | × |
| **WAT** | × | × | × | × | × | × | × | ↑ | ↑ | ○ | ○ | ○ |

“↑” indicates that land cover change promotes NPP increase, “↓”indicates that land cover change promotes NPP reduction, “×” indicates an illogical result in terms of ground cover change or a scenario that will not happen in the short term, and “○” indicates that land cover change promotes little change in NPP.

For two consecutive years, the reduction in NPP is recorded as NPP (↓), i.e., *NPP_i + 1_*<*NPP_i_*, and the increase is recorded as NPP (↑), i.e., *NPP_i + 1_*>*NPP_i_* (Eq 10). The total decrease or increase in NPP from 2001 to 2015 is recorded as *S_↓NPP_* or *S_↑NPP_*, respectively (Eq 11). The total decrease in NPP caused by drought or drought intensification and the increase caused by drought relief over 15 years are recorded as *S_↓D_NPP_* and S*_↑D_NP_*_P_, respectively (Eq 12). The total decrease or increase in NPP caused by the overlap area of drought and LCC is recorded as *S_↓D_LCC_NPP_* and *S_↑D_LCC_NPP_,* respectively (Eq 13). The total contribution of drought to the decrease or increase in NPP from 2001 to 2015 is recorded as *SCR_↓D_NPP_* and *SCR_↑D_NPP_*_,_ respectively (Eq 14).

*NPP_↓↑_*=$\left\{ \begin{aligned} NPP\left( \downarrow\right)=\downarrow\left( {NPP}_{i+1}-{NPP}_{i} \right), ({NPP}_{i+1}<{NPP}_{i}) \\ NPP\left( \uparrow\right)=\uparrow\left( {NPP}_{i+1}-{NPP}_{i} \right), ({NPP}_{i+1}>{NPP}_{i}) \end{aligned} \right.$ (10)

*S_↓↑(NPP)_*$=$ $\left\{ \begin{aligned} S_{\downarrow NPP}=\sum_{i=2001}^{2015} \left( \left. \downarrow\left( {NPP}_{i+1}-{NPP}_{i} \right) \right), ({NPP}_{i+1}<{NPP}_{i}) \right. \\ S_{\uparrow NPP}=\sum_{i=2001}^{2015} \left( \left. \uparrow\left( {NPP}_{i+1}-{NPP}_{i} \right) \right), \right. ({NPP}_{i+1}>{NPP}_{i}) \end{aligned} \right.$ (11)

*S_↓↑(D_NPP)_*$=$ $\left\{ \begin{aligned} S_{\downarrow D\_NPP}=\sum_{i=2001}^{2015} \left( \left. \downarrow\left( {NPP}_{i+1}-{NPP}_{i} \right)*D \right), ({NPP}_{i+1}<{NPP}_{i}) \right. \\ S_{\uparrow D\_NPP}=\sum_{i=2001}^{2015} \left( \left. \uparrow\left( {NPP}_{i+1}-{NPP}_{i} \right)*N \right), \right. ({NPP}_{i+1}>{NPP}_{i}) \end{aligned} \right.$ (12)

*S_↓↑(D__LCC_NPP)_*$=$ $\left\{ \begin{aligned} S_{\downarrow D\_LCC\_NPP}=\sum_{i=2001}^{2015} \left( \left. \downarrow\left( {NPP}_{i+1}-{NPP}_{i} \right)*D*R_{LCC (\downarrow)} \right), ({NPP}_{i+1}<{NPP}_{i}) \right. \\ S_{\uparrow D\_\_LCCNPP}=\sum_{i=2001}^{2015} \left( \left. \uparrow\left( {NPP}_{i+1}-{NPP}_{i} \right)*N*I_{LCC (\uparrow)} \right) \right., ({NPP}_{i+1}>{NPP}_{i}) \end{aligned} \right.$ (13)

*SCR_↓↑(D_NPP)_*_=_$\left\{ \begin{aligned} \frac{S_{\downarrow D\_NPP}-S_{\downarrow D\_LCC\_NPP}}{S_{\downarrow NPP}}*100\%, ({NPP}_{i+1}<{NPP}_{i}) \\ \frac{S_{\uparrow D\_NPP}-S_{\uparrow D\_\_LCCNPP}}{S_{\uparrow NPP}}*100\%, ({NPP}_{i+1}>{NPP}_{i}) \end{aligned} \right.$ (14)

Similarly, *S_↓LCC_NPP_* and *S_↑LCC_NPP_* indicate the total decrease or increase in NPP caused by LCC, respectively (Eq 15); *SCR_↓LCC_NPP_* and *SCR_↑LCC_NPP_* indicate the total contribution of LCC to the decrease or increase in NPP, respectively (Eq 16); *S_↓SPEI(d)_NPP_,S_↑SPEI(I)_NPP_* indicate the total decrease or increase in NPP caused by hydrothermal conditions, respectively (Eq 17); *S_↓D_LCC_NPP_* and *S_↑D_LCC_NPP_* indicate the total decrease or increase in NPP caused by the overlap area of drought and LCC, respectively (Eq 18); and *SCR_↓SPEI_NPP_* and *SCR_↑SPEI_NPP_* indicate the total contribution of hydrothermal conditions to the decrease or increase in NPP, respectively (Eq 19).

*S_↓↑(LCC_NPP)_*$=$ $\left\{ \begin{aligned} S_{\downarrow LCC\_NPP}=\sum_{i=2001}^{2015} \left( \left. \downarrow\left( {NPP}_{i+1}-{NPP}_{i} \right)*R_{LCC (\downarrow)} \right), ({NPP}_{i+1}<{NPP}_{i}) \right. \\ S_{\uparrow LCC\_NPP}=\sum_{i=2001}^{2015} \left( \left. \uparrow\left( {NPP}_{i+1}-{NPP}_{i} \right)*I_{LCC (\uparrow)} \right), \right. ({NPP}_{i+1}>{NPP}_{i}) \end{aligned} \right.$ (15)

*SCR_↓↑(LCC_NPP)=_*$\left\{ \begin{aligned} \frac{S_{\downarrow LCC\_NPP}}{S_{\downarrow NPP}}*100\%, ({NPP}_{i+1}<{NPP}_{i}) \\ \frac{S_{\uparrow LCC\_NPP}}{S_{\uparrow NPP}}*100\%, ({NPP}_{i+1}>{NPP}_{i}) \end{aligned} \right.$ (16)

*S_↓↑(SPEI_NPP)_*$=$ $\left\{ \begin{aligned} S_{\downarrow SPEI(d)\_NPP}=\sum_{i=2001}^{2015} \left( \left. \downarrow\left( {NPP}_{i+1}-{NPP}_{i} \right)*SPEI (d) \right), ({NPP}_{i+1}<{NPP}_{i}) \right. \\ S_{\uparrow SPEI(I)\_NPP}=\sum_{i=2001}^{2015} \left( \left. \uparrow\left( {NPP}_{i+1}-{NPP}_{i} \right)*SPEI (I) \right), \right. ({NPP}_{i+1}>{NPP}_{i}) \end{aligned} \right.$ (17)

*S_↓↑(SPEI__LCC_NPP)_*$=$ $\left\{ \begin{aligned} S_{\downarrow SPEI\_LCC\_NPP}=\sum_{i=2001}^{2015} \left( \left. \downarrow\left( {NPP}_{i+1}-{NPP}_{i} \right)*SPEI(d)*R_{LCC (\downarrow)} \right), ({NPP}_{i+1}<{NPP}_{i}) \right. \\ S_{\uparrow SPEI\_\_LCCNPP}=\sum_{i=2001}^{2015} \left( \left. \uparrow\left( {NPP}_{i+1}-{NPP}_{i} \right)*SPEI(I)*I_{LCC (\uparrow)} \right) \right., ({NPP}_{i+1}>{NPP}_{i}) \end{aligned} \right.$(18)

*SCR_↓↑(SPEI_NPP)=_*$\left\{ \begin{aligned} \frac{S_{\downarrow SPEI\_NPP}-S_{\downarrow SPEI\_LCC\_NPP}}{S_{\downarrow NPP}}*100\%, ({NPP}_{i+1}<{NPP}_{i}) \\ \frac{S_{\uparrow SPEI\_NPP}-S_{\uparrow SPEI\_\_LCC\_NPP}}{S_{\uparrow NPP}}*100\%, ({NPP}_{i+1}>{NPP}_{i}) \end{aligned} \right.$ (19)

## 2.5 Quantifying the contribution of major land use change to NPP

According to China's land use classification system, cropland (CRO) and cropland/natural vegetation mosaic (CNV) are merged into cropland; evergreen needleleaf forest (ENF), deciduous broadleaf forest (DBF), mixed forest (MF), closed shrublands (CSH), open shrublands (OSH), woody savannas (WSA) are merged into forest; water (WAT) and permanent wetlands (WET) are merged into water area; and grasslands (GRA), urban and built-up (URB), and barren or sparsely vegetated (BSV) are defined as grassland, urban land, and unused land, respectively. Table 3 shows the land use transfer matrix of the Loess Plateau from 2001 to 2015. The main land use types (cropland, forest land, and grassland) that produced NPP changed drastically and had the greatest impact on NPP. We focus on quantifying the contribution of these three land uses to each other or to non-NPP production land use (water area, urban land, unused land) to the changes in NPP. *R__LUCC(↓_*_)_ and *I__LUCC(↑)_* indicate the area where the land use change caused NPP to decrease or increase, respectively (Eqs 20 and 21)); *S_↓LUCC_NPP_* and *S_↑LUCC_NPP_* (Eq 22) indicate that land use type conversion caused NPP to decrease or increase, respectively; and *SCR_↓LUCC_NPP_* and *SCR_↑LUCC_NPP_* (Eq 23) represent the total contribution of land use type conversion to a reduction or increase in NPP, respectively.

*R__LUCC (↓)_*=$\left\{ \begin{aligned} 1\left\{ \begin{aligned} \mathrm{LUCC}\left( \left. \mathrm{FOR} \right) \right.to LUCC\left( \left. CRO,GRA,CRI,WAT,UNU \right) \right. \\ or LUCC\left( \mathrm{CRO} \right.)to LCC\left( \left. GRA,CRI,WAT,UNU \right) \right. \\ \mathrm{orLCC}\left( \left. \mathrm{GRA} \right) \right.to LCC\left( \left. CRI,WAT,UNU \right) \right. \end{aligned} \right. \\ 0 or else \end{aligned} \right.$ (20)

*I__LUCC (↑)_*=$\left\{ \begin{aligned} 1\left\{ \begin{aligned} \mathrm{LCC}\left( \left. \mathrm{CRO} \right) \right.to LCC\left( \left. \mathrm{FOR} \right) \right. \\ or LCC\left( \left. \mathrm{GRA} \right) \right.to LCC\left( \left. CRO,FOR \right) \right. \\ \end{aligned} \right. \\ 0 or else \end{aligned} \right.$ (21)

In Eqs 20 and 21, FOR, CRO, GRA, WAT, CRI, and UNU represent cropland, forest, grassland, water area, urban land, and unused land, respectively.

*S_↓↑(LUCC_NPP)_*$=$ $\left\{ \begin{aligned} S_{\downarrow LUCC\_NPP}=\sum_{i=2001}^{2015} \left( \left. \downarrow\left( {NPP}_{i+1}-{NPP}_{i} \right)*R_{LUCC (\downarrow)} \right), ({NPP}_{i+1}<{NPP}_{i}) \right. \\ S_{\uparrow LUCC\_NPP}=\sum_{i=2001}^{2015} \left( \left. \uparrow\left( {NPP}_{i+1}-{NPP}_{i} \right)*I_{LUCC (\downarrow)} \right), \right. ({NPP}_{i+1}>{NPP}_{i}) \end{aligned} \right.$ (22)

*SCR_↓↑(LUCC_NPP)=_*$\left\{ \begin{aligned} \frac{S_{\downarrow LUCC\_NPP}}{S_{\downarrow NPP}}*100\%, ({NPP}_{i+1}<{NPP}_{i}) \\ \frac{S_{\uparrow LUCC\_NPP}}{S_{\uparrow NPP}}*100\%, ({NPP}_{i+1}>{NPP}_{i}) \end{aligned} \right.$ (23)

**Table 3. Land use transfer matrix for 2001-2015 on the Loess Plateau (km^2^)**

|  | **city** | **cropland** | **forest** | **grassland** | **unused_land** | **water** |
| --- | --- | --- | --- | --- | --- | --- |
| **city** | 10564.3 | 619.0 | 130.3 | 265.3 | 14.7 | 0.7 |
| **cropland** | 270.3 | 121218.0 | 7207.6 | 24297.5 | 19.5 | 0.8 |
| **forest** | 15.1 | 4065.6 | 50849.6 | 12260.2 | 139.3 | 41.5 |
| **grassland** | 88.0 | 7006.2 | 3500.0 | 359925.0 | 5259.5 | 22.3 |
| **unused_land** | 3.7 | 25.4 | 4.8 | 820.0 | 14360.8 | 13.4 |
| **water** | 0.2 | 37.4 | 144.1 | 202.1 | 43.4 | 447.3 |

## 2.6 Quantifying the contribution of different degrees of drought aggravation or mitigation to NPP changes

To clarify the impact of different drought severities or reductions on NPP, we divided the drought level into 5 grades (Table 1). The frequency of light drought was 100%, the frequency of moderate drought was 93%, and the average area percentage of light drought or moderate drought was 76% (Fig 4). We calculated the contribution of NPP change caused by the aggravation or alleviation of light and moderate drought.

We recorded the areas where light drought was aggravated or mitigated for two consecutive years as *A__LD_* and *R__LD_*, respectively (Eqs 24 and 25); additionally, the areas where moderate drought was aggravated or mitigated for two consecutive years were recorded as *A__MD_* and *R__MD_*, respectively (Eqs 26 and 27). The total amount of changes in NPP caused by the aggravation or mitigation of drought in areas where light drought occurs is recorded as *S_↓LD_NPP_* and *S_↑LD_NPP_*, respectively (Eq 28). The total amount of changes in NPP caused by the aggravation or mitigation of drought in areas where moderate drought occurs is recorded as *S_↓MD_NP_*_P_ and *S_↑MD_NPP_*, respectively (Eq 29), and the combined effects of light or moderate drought and LCC causing the total change in NPP were recorded as *S_↓LD_LCC_, S_↓MD_LCC_, S_↑LD_LCC_* and *S_↑MD_LC_*_C_, respectively (Eqs 30 and 31). We also recorded the total contribution of changes in NPP caused by the aggravation or mitigation of light or moderate drought as *SCR_↓LD_NPP_*, *SCR_↓MD_NPP_, SCR_↑LD_NPP_*, and *SCR_↑MD_NPP_* (Eqs 32 and 33)

*A__LD_*=$\left\{ \begin{aligned} 1 \left( \left( \left. {SPEI}_{i}>\left( \left. -0.5 \right) \right.and(-1)<{SPEI}_{i+1}\leq\left( \left. -0.5 \right) \right. \right) \right.\mathrm{or}\left( (-1)<\left. {SPEI}_{i+1}<{SPEI}_{i}\leq\left( -0.5 \right) \right) \right. \right) \\ 0 or else \end{aligned} \right.$ (24)

*R__LD_=*$\left\{ \begin{aligned} 1 \left( \left( \left. (-1)<{SPEI}_{i}\leq\left( \left. -0.5 \right)and \right.{SPEI}_{i+1}>\left( \left. -0.5 \right) \right. \right) \right.or\left( (-1)<\left. {SPEI}_{i}<{SPEI}_{i+1}\leq\left( -0.5 \right) \right) \right. \right) \\ 0 or else \end{aligned} \right.$ (25)

*A__MD_*=$\left\{ \begin{aligned} 1 \left( \left( \left. {SPEI}_{i}>\left( \left. -1 \right) \right.and(-1.5)<{SPEI}_{i+1}\leq\left( \left. -1 \right) \right. \right) \right.or\left( (-1.5)<\left. {SPEI}_{i+1}<{SPEI}_{I}\leq\left( -1 \right) \right) \right. \right) \\ 0 or else \end{aligned} \right.$ (26)

*R__MD_*=$\left\{ \begin{aligned} 1 \left( \left( \left. \left( -1.5 \right)<{SPEI}_{i}\leq\left( \left. -1 \right) \right.and {SPEI}_{i+1}>\left( \left. -1 \right) \right. \right) \right.or\left( (-1.5)<\left. {SPEI}_{i}<{SPEI}_{i+1}\leq\left( -1 \right) \right) \right. \right) \\ 0 or else \end{aligned} \right.$ (27)

*S_↓↑(LD_NPP)_*$=$ $\left\{ \begin{aligned} S_{\downarrow{ED\_}_{NPP}}=\sum_{i=2004}^{2005} \left( \left. \downarrow\left( {NPP}_{i+1}-{NPP}_{i} \right)*A_{\_LD} \right), \left( {NPP}_{i+1}<{NPP}_{i} \right) \right. \\ S_{\uparrow{ED\_}_{NPP}}=\sum_{i=2005}^{2006} \left( \left. \uparrow\left( {NPP}_{i+1}-{NPP}_{i} \right)*R_{\_LD} \right), \right. \left( {NPP}_{i+1}>{NPP}_{i} \right) \end{aligned} \right.$ (28)

*S_↓↑(MD_NPP)_*$=$ $\left\{ \begin{aligned} S_{\downarrow{ED\_}_{NPP}}=\sum_{i=2004}^{2005} \left( \left. \downarrow\left( {NPP}_{i+1}-{NPP}_{i} \right)*A_{\_MD} \right), \left( {NPP}_{i+1}<{NPP}_{i} \right) \right. \\ S_{\uparrow{ED\_}_{NPP}}=\sum_{i=2005}^{2006} \left( \left. \uparrow\left( {NPP}_{i+1}-{NPP}_{i} \right)*R_{\_MD} \right), \right. \left( {NPP}_{i+1}>{NPP}_{i} \right) \end{aligned} \right.$ (29)

*S_↓↑(LD_LCC)_*$=$ $\left\{ \begin{aligned} S_{\downarrow LD\_LCC}=\sum_{i=2004}^{2005} \left( \left. \downarrow\left( {NPP}_{i+1}-{NPP}_{i} \right)*A_{\_LD}*R_{LCC (\downarrow)} \right), ({NPP}_{i+1}<{NPP}_{i}) \right. \\ S_{\uparrow LD\_\_LCC}=\sum_{i=2005}^{2006} \left( \left. \uparrow\left( {NPP}_{i+1}-{NPP}_{i} \right)*R_{\_LD}*I_{LCC (\uparrow)} \right) \right., ({NPP}_{i+1}>{NPP}_{i}) \end{aligned} \right.$ (30)

*S_↓↑(MD_LCC)_*$=$ $\left\{ \begin{aligned} S_{\downarrow LD\_LCC}=\sum_{i=2004}^{2005} \left( \left. \downarrow\left( {NPP}_{i+1}-{NPP}_{i} \right)*A_{\_MD}*R_{LCC (\downarrow)} \right), ({NPP}_{i+1}<{NPP}_{i}) \right. \\ S_{\uparrow LD\_\_LCC}=\sum_{i=2005}^{2006} \left( \left. \uparrow\left( {NPP}_{i+1}-{NPP}_{i} \right)*R_{\_MD}*I_{LCC (\uparrow)} \right) \right., ({NPP}_{i+1}>{NPP}_{i}) \end{aligned} \right.$ (31)

*SCR_↓↑(LD_NPP)=_*$\left\{ \begin{aligned} \frac{S_{\downarrow LD\_NPP}-S_{\downarrow LD\_LCC}}{S_{\downarrow NPP}}*100\%, ({NPP}_{i+1}<{NPP}_{i}) \\ \frac{S_{\uparrow LD\_NPP}-S_{\uparrow LD\_\_LCC}}{S_{\uparrow NPP}}*100\%, ({NPP}_{i+1}>{NPP}_{i}) \end{aligned} \right.$ (32)

*SCR_↓↑(MD_NPP)=_*$\left\{ \begin{aligned} \frac{S_{\downarrow MD\_NPP}-S_{\downarrow MD\_LCC}}{S_{\downarrow NPP}}*100\%, ({NPP}_{i+1}<{NPP}_{i}) \\ \frac{S_{\uparrow MD\_NPP}-S_{\uparrow MD\_\_LCC}}{S_{\uparrow NPP}}*100\%, ({NPP}_{i+1}>{NPP}_{i}) \end{aligned} \right.$ (33)

**Fig 4. Percentage of the area of light or moderate drought to the total area of drought.**

LD_D_A indicates the percentage of the area where light drought occurred to the area where drought occurred (%), MD_D_A indicates the percentage of the area where the moderate drought occurred in the area where the drought occurred (%), and LD_MD_A (%) indicates the total proportion of the area with light or moderate drought to the total area where drought occurred.

# Results and Analysis

## 3.1 Interannual changes in NPP and the SPEI from 2000 to 2015

### 3.1.1 Distribution and changing trend of NPP

The average value of NPP in the Loess Plateau over the past 15 years was generally low (Fig 5) and showed a decreasing trend from southeast to northwest. The low-value NPP area (0-200 gC/m^2^) was widely distributed and concentrated in the gully area of the western plateau, the desert area in the north-central sandy land, the Hetao Plain and some hilly gully areas in the north. The vegetation was sparse, and the surface cover was mostly grassland or cropland. High-value areas (600-800 gC/m^2^) were concentrated in the mountains and valley plains in the south and east. The median range (200-600 gC/m^2^) was between the high and low.

The linear variation of NPP in most areas of loess plateau showed an increasing trend. The areas where the NPP growth rate was fast were concentrated in part of the cropland in the western gully, the central Hetao Plain, the central Loess Plateau and southern valley plain area, with an average annual growth rate of >40 gCm^-2^ year^-1^, and the growth of some areas was >50 gCm^-2^ year^-1^ (Fig 6A). The land cover was mainly cropland. The areas where the NPP growth rate was slow were concentrated in the Middle East and the northern edge of the Loess Plateau, with an average annual growth rate of <20 gCm^-2^ year^-1^. The land cover was mainly grassland. The sporadic distribution of forest area in the south central region had the highest average NPP value for many years and primarily showed a downward trend over the past 15 years, with an average annual decrease of <30 gCm^-2^ year^-1^. The annual average NPP in some areas of Ziwuling, Luliang Mountain and other areas decreased by >40 gCm^-2^year^-1^. Fig 6B shows that NPP changed significantly in the western, central north, Guanzhong Plain, and eastern Fenhe Valley of the Loess Plateau over the past 15 years, with an average annual increase rate of >30%. The land cover was mainly grassland and cropland.

**Fig 5. Average NPP (g C/m^2^) of the Loess Plateau from 2000 to 2015.**

**Fig 6**. **Linear change in NPP on the Loess Plateau from 2000 to 2015.** (a) NPP linear change trend (gCm^-2^ year^-1^).(b) NPP linear change rate (%).

### 3.1.2 Changes in the SPEI and correlation with NPP

The SPEI of the Loess Plateau varied from -0.1 to 0.15 over the past 15 years, and 76% of the regions showed an increase in the SPEI. Drought in the study area was generally alleviated (Fig 7A). Figs 6A and 7A show that the growth rate of SPEI and NPP is relatively fast in the agricultural irrigation area of the Hetao Plain in the north, Taihang Mountain in the northeast and areas of the Loess Plateau in the middle. The relief of drought and changes in hydrothermal conditions were important factors affecting the increase in NPP. A significant correlation was observed between the SPEI and NPP (p<0.1). Approximately 81.3% of the areas showed a positive correlation with the SPEI (Fig 7B). The SPEI and NPP were extremely significantly positively correlated (p<0.05) in the northeast of the Taihang Mountains, the Loess Plateau, with Yulin and Yan'an as the center, and part of the plateau gully in the west-central area. The land cover was mostly grassland or cropland.

Approximately 18.6% of the areas showed a negative correlation between the SPEI and NPP, and these areas were mainly distributed in the forest of the gully in the western plateau, the south-central mountain in the middle, part of the cropland in the Guanzhong Plain, and part of the cropland and grassland in the eastern margin. The SPEI in the forest land showed an increasing trend while NPP showed a downward trend; in contrast, the SPEI in the cropland or grassland showed a decreasing trend while NPP showed an increasing trend. Human factors were the main factors affecting the change in NPP in these regions.

**Fig** **7. Correlation between the SPEI and NPP in the Loess Plateau from 2000 to 2015.** (a) Linear trend of the SPEI. (b) Correlation coefficient between the SPEI and NPP.

## **3.2 Contribution of hydrothermal conditions to NPP changes**

Fig 8A shows that the contribution of hydrothermal changes to the reduction of NPP is approximately 61% on average, and the area where the contribution exceeds 80% is distributed in the northeast, southwest, central, and eastern part of the Fenhe Valley in the Loess Plateau. The land cover of these areas is grassland or cropland. Fig 8B shows that the contribution of hydrothermal change to the increase in NPP is approximately 59% on average. The regions where the contribution exceeds 80% partially coincide with the high value area for NPP reduction. Precipitation and evapotranspiration are the main factors for changes of NPP.

**Fig 8. Contribution (%) of hydrothermal conditions to NPP on the Loess Plateau from 2001 to 2015 (%).** (a) Contribution (%) of hydrothermal change to NPP reduction. (b) Contribution of hydrothermal condition mitigation to NPP increases (%).

## 3.3 Contribution of drought to NPP changes

### 3.3.1 Contribution of drought aggravation or mitigation to NPP changes

Fig 9A shows that the contribution of drought or drought aggravation to the reduction of NPP was approximately 33%. High value areas (>40%) were evenly and widely distributed. The contribution of some grassland in the central hilly gully region, plateau-gully region, and part of the cropland in Guanzhong Plain south of the central part was the highest (>80%). The bold text in Table 4 indicates that the average annual reduction of NPP in eight consecutive years was much greater and the area percentage of the arid area in seven years was similarly large on the Loess Plateau. Although the percentage of arid area in the remaining year was not large, the drought in the arid regions rapidly intensified over 13-14 years, and the aggravation caused the area to transition from no drought to severe drought. Thus, drought was an important factor affecting the reduction of NPP.

The contribution of drought mitigation to the increase in NPP was approximately 32%. Fig 9B shows that the contribution in most areas was greater than 40%. NPP extending from the northeast to a part of the west was most sensitive to the response to drought mitigation, and its contribution was greater than 70%. In particular, the contribution of the hilly gully region in the northeast, Taihang Mountain in the east and gully in the western plateau was more than 80%. The aboveground cover was mostly grassland, and it had a higher contribution to the increase in NPP. In addition, the high contribution areas (>50%) of the central and eastern regions coincided with the rapid growth of NPP in the 15-year period. The relief of drought was an important factor affecting the increase in NPP.

**Table 4. Arid area percentage and average NPP reduction**

| **Year** |  | **Drought area (%)** | **D_NPP (g/m^2^)** |
| --- | --- | --- | --- |
| 01_02 |  | 12 | 5 |
| 02_03 |  | 0 | 4 |
| **03_04** |  | **31** | **20** |
| **04_05** |  | **60** | **21** |
| 05_06 |  | 29 | 9 |
| 06_07 |  | 10 | 7 |
| **07_08** |  | **42** | **18** |
| **08_09** |  | **49** | **17** |
| 09_10 |  | 14 | 9 |
| **10_11** |  | **29** | **44** |
| 11_12 |  | 15 | 7 |
| **12_13** |  | **30** | **24** |
| **13_14** |  | 8 | **31** |
| **14_15** |  | **35** | **24** |

Year indicates two consecutive years, e.g., 01_02 indicates 2001-2002; drought area indicates the percentage of drought area; and D_NPP indicates the average reduction in NPP between two consecutive years.

**Fig 9. Drought contribution to NPP on the Loess Plateau from 2001 to 2015 (%).** (a) Contribution of drought to NPP reduction (%). (b) Contribution of drought mitigation to NPP increase (%).

### 3.3.2 Contribution of different levels of drought to NPP changes

To help local governments maintain regional ecosystem stability and security, we quantified the contribution of NPP reduction caused by the aggravation of drought to light or moderate drought (Fig 10). The contribution of NPP associated with the relief of light or moderate drought increased on the prefecture and city scales (Fig 11). The impact of aggravating or mitigating light drought on NPP was stronger than that of moderate drought. Fig 10A shows the contribution of NPP reduction from no drought to light drought and indicates that the degree of light drought will be aggravated within the same drought level at an average of 13%. High values (>20%) were distributed in the gully area of the mid-west, and the land cover was mostly grassland and cropland. In addition, 6 of the 44 cities had a contribution of >20%, and Yan'an was the highest (23.1%). Fig 10B shows the contribution of NPP reduction associated with no drought or light drought to moderate drought, and it shows that the degree of moderate drought will be aggravated within the same drought level at an average of 10%. The high-value area (>20%) was distributed in the western plateau gully area and the central edge of the agricultural irrigation area, and the land cover was mostly grassland. In addition, 4 of the 44 cities had a contribution of >20%, and Linxia was the highest (28%).

Fig 11 shows the contribution of the increase in NPP caused by the alleviation of light or moderate drought. The contribution of NPP increase from light drought to no drought and the degree of light drought will be alleviated within the same drought level at an average of 12.7%. The high-value area (>20%) was distributed in the western marginal gully area and the central gully or plain area. In addition, 6 of the 44 cities had a contribution of >20%, and Hainan Tibetan was the highest (41%) (Fig 11A). The average contribution to the increase in NPP was 9.4%, in which drought was alleviated from moderate drought to light drought or no drought and the degree of moderate drought was alleviated within the same drought level. High-value areas (>20%) were distributed in the gully area of the western plateau, and the land cover was mostly grassland. In addition, 2 of the 44 cities had a contribution of>20%, and Baiyin was the highest (32%) (Fig 11B).

**Fig 10. Contribution of NPP reduction caused by drought aggravation in 2001-2015 at the prefecture level on the Loess Plateau.** (a) Contribution of NPP reduction caused by the drought changes from no drought to light drought and the degree of light drought increased within the same drought level. (b) Contribution of NPP reduction caused by the drought changing from no drought or light drought to moderate drought, and the degree of moderate drought increased within the same drought level.

**Fig 11. Contribution to NPP increase caused by drought relief in 2001-2015 at the prefecture level on the Loess Plateau.** (a) Contribution to NPP increase caused by drought relief from light drought to no drought, and the degree of light drought has been alleviated within the same drought level. (b) Contribution to NPP increase caused by drought relief from moderate drought to light drought or no drought, and the degree of moderate drought has been alleviated within the same drought level.

## 3.4 Contribution of land cover change (LCC) to NPP changes

The contribution of LCC to the decrease or increase of NPP was generally smaller than that of drought to the change of NPP, and the spatial distribution was uneven (Figs 12A-B). The areas where LCC caused a reduction of NPP were concentrated in the south-central gully region and the Taihang Mountain area in the east, and the land cover was forest land, such as Ziwu Mountain, Qinling Mountains in the middle and Luliang Mountain, Taihang Mountain in the east. This result is consistent with the linear decrease in NPP shown in Fig 6. The contribution to the reduction in NPP caused by LCC was generally between 5% and 10%, with an average of approximately 1.4%. The change in land cover was mainly reflected by the conversion of forest land to others. The contribution of some forest land in Ziwuling in the middle was between 25% and 30%, and the contribution of some forest land in Luliang Mountain was greater than 60%. Areas where LCC contributed to the increase in NPP were concentrated in the cropland or grassland around the south-central and eastern forest. The contribution was generally concentrated between 5% and 10%, with an average of approximately 0.5%. The change of land cover was mainly reflected by the conversion from grassland or cropland to cropland or forest land. The contribution of some grassland areas on the northern border of Zhongwei and Wuzhong was higher than 50%.

**Fig 12.** **Contribution of LCC to NPP on the Loess Plateau from 2001 to 2015 (%).** (a) Contribution of LCC to NPP reduction (%). (b) Contribution of LCC to NPP increase (%).

### 3.4.1 Contribution of major land use change to NPP changes

To help the local governments establish a healthy ecosystem, we focused on quantifying the contribution of the main land uses (forest land, cropland and grassland) to each other or to non-NPP major production land uses (cities, water, and unused land) to NPP on a city-level scale. Figs 13A-C show the average contribution of the reduction of NPP caused by the conversion of forest land, cropland and grassland in each city. The contribution was relatively low, i.e., below 5%. Fig 13A shows the contribution of the conversion of forest land to non-forest land to the reduction of NPP, and it had an average of approximately 0.2%. The high-value areas were mainly distributed in the Taihang Mountain area and valley plain in the southeast and south-central regions. In addition, 5 of the 44 cities had a contribution of >0.8%, and Jiyuan had the highest contribution (1.6%). Fig 13B shows the conversion of cropland to grassland or non-NPP main production land that contributed to the reduction of NPP, and it had an average of approximately 0.3%. The high-value areas were mainly distributed in the northern Hetao Plain, the western plateau gully and the eastern Fenhe Valley, and Yangquan had the highest value (0.84%). Fig 13C shows the contribution of NPP reduction caused by grassland conversion to non-NPP main production land, and it had an average of approximately 0.04%, and Wuhai was the highest (1.3%).

Figs 14A-B show the average contribution of the increase in NPP caused by the conversion of cropland or grassland to forest land or cropland in each city. The contribution was generally low, i.e., less than 5%. The average contribution to the increased NPP by the conversion of cropland to forest land was approximately 0.1%. The high value areas were mainly distributed in Guanzhong Plain in the south of the central part and Fenhe Valley in the East. In addition, 3 of the 44 cities had a contribution of >0.8%, and Tongchuan had the highest value (1.4%) (Fig 14A). The average contribution of NPP increase caused by grassland conversion to cropland or forest land is 1.1%. In addition, 20 of the 44 cities had the contribution of >0.8%, and Linfen had the highest value (4%) (Fig 14B). Among the three land uses, the conversion of cropland to grassland or non-NPP main production land use had the greatest impact on the reduction of NPP, and the conversion of grassland to cropland or forest land had the greatest impact on the increase in NPP.

**Fig 13. Contribution of land use change to NPP reduction on the Loess Plateau in 2000-2015 (%).** (a) Contribution of NPP reduction caused by conversion of forest land to non-forest land (%). (b) Contribution of NPP reduction caused by cropland conversion to grassland or non-NPP main production land (%). (c) Contribution of NPP reduction caused by grassland conversion to non-NPP main production land (%).

**Fig 14. Contribution of land use change to the increase in NPP on the Loess Plateau from 2001 to 2015 (%).** (a) Contribution of conversion of cropland to forest land to the increase in NPP (%). (b) Contribution of grassland conversion to cropland or forest land to the increase in NPP (%).

# Discussion

## 4.1 Accuracy of NPP from its estimation model

The major error of NPP estimates are associated with the parameter selection and construction methods of different models. The light energy utilization model we adopted based on the GLM_PEM is a productivity model that is mainly driven by remote sensing data. This model can make full use of the advantages of remote sensing data to produce high-resolution estimates of regional-scale vegetation, especially for forest land NPP [[20](#_ENREF_20)]. Liang et al. performed a correlation analysis on the NPP values estimated by this model and the observations of 51 gross primary production (GPP) flux tower stations around the world. The correlation coefficient R obtained here was higher and better than that obtained by the CASA model, which verified the accuracy of the model in estimating NPP [[27](#_ENREF_27)]. At present, the GLM_PEM model has been successfully applied to NPP and GPP estimation studies of global terrestrial ecosystems [[28-31](#_ENREF_28)]. Therefore, we used this model to ensure the accuracy of the NPP data.

## 4.2 Advantages and limitations of the research methods

NPP change is a complex process with many influencing factors, including drought, land use, topographical factors, climatic factors, human activities and other factors. Scholars have studied the influencing factors of NPP from different perspectives [[32-34](#_ENREF_32)]. In this study, we mainly quantify the impact of drought and land use change to NPP. Our research shows that ~61% of NPP reduction was affected by hydrothermal conditions, ~33% was affected by drought, ~13% was affected by the aggravation of drought to light or medium drought and ~1.4% was affected by LCC. Moreover, ~59% of the NPP increase was affected by hydrothermal conditions, ~32% was affected by drought relief, ~12.7% was affected by the alleviation of light or moderate drought, and ~1.1% was affected by LCC. In addition, we also specifically studied the contribution of major land use change to NPP variations. The results are accurate and the mechanisms underlying the effects of drought and land use change on NPP are clarified, thus providing insights on effective methods of building a healthy ecosystem. The main factors of NPP change in overlapping areas are drought and LCC. In this study, overlapping areas generally show the influence of LCC on NPP, while the contribution of drought to NPP is underestimated. This limitation is one of the difficulties involved in optimizing the measurement method and should be addressed in the future.

## 4.3 Differentiating the effects of hydrothermal conditions, drought, and land cover change (LCC) on NPP variations

We found that hydrothermal conditions had the strongest impact on NPP, followed by drought. The impact of LCC on NPP variations was relatively small; however, the contribution of LCC to variations in NPP was relatively high (>50%) in some regions, which is because the influence of hydrothermal conditions and drought on NPP mainly shows a "planar" distribution in space, i.e., these two factors mainly affect the change in NPP over a large range, while the influence of LCC on NPP is mainly shows a "point" distribution in space, i.e., LCC mainly affects the variations in local NPP. Although the impact of LCC on NPP is relatively low on average, its contribution in some areas is relatively large and shows values greater than 80%. This result is because LCC mainly occurs locally; thus, in some small areas, LCC is the leading factor affecting NPP change.

## 4.4 Measuring the contribution of major land use change to NPP variations reflects the change of ecological environment

The ecological environment of the Loess Plateau is fragile; however, ecological protection measures, such as China's GTGP and Land Desertification Control Project (LDCP), have been implemented since 1990, and these programs have restored the ecological environment in certain areas [[35](#_ENREF_35)]. NPP can be used to assess the production capacity and sustainable development of ecosystems. In this study, we quantified the contribution of major land-use change to variations in NPP reflected in changes in the ecological environment of the Loess Plateau over the past 15 years. The contribution of the main land use change to the increase in NPP reflects the effect of ecological environment restoration. The results show that in some areas, the conversion of cropland to forest land is the main factor that increased NPP. The GTGP and LDCP are effective methods of promoting NPP growth. Research shows that most of the cropland in Baoji was converted into forest, and some of the cropland contributed more than 25% to the increase in NPP; moreover, the values were greater than 45% in the south, which reflected the good effect of returning cropland to forest (Fig 15A). The forest coverage of Baoji has increased from 45% to 51% (675 km^2^), the cropland coverage has decreased from 47% to 44% (278 km^2^), and the NPP has increased by 11 gCm^-2^ year^-1^ in the 15-year period. These results were consistent with the research trend shown by Xu Yuxia for the return of farmland to forest from 1999 to 2015 for Baoji City [[36](#_ENREF_36)].

Research shows that the contribution of NPP increase from the conversion of wasteland and sandy land to forest land, grassland and cropland in Baiyin is high and generally higher than 45% (Fig 15B). The wasteland and sandy land in this city have been reduced by 407 km^2^, with the coverage rate reducing from 5% to 3%, and the forest land has been increased by 268 km^2^, with the coverage rate increasing from 1% to 3%. Moreover, NPP has increased by 14 gCm^-2^ year^-1^. Thus, the GTGP and LDCP can be implemented to control ecological environmental problems and promote an increase in NPP to ensure the safety and stability of the ecosystem.

**Fig 15. Contribution of conversion from cropland to forest land to NPP increase.** (a) Contribution of NPP increase from cropland to forest land. (b) Contribution of NPP increase from unused land to grassland, forest land and cropland.

# Conclusions

We analyzed the temporal and spatial change characteristics of NPP on the Loess Plateau from 2000 to 2015 and quantified the contributions of hydrothermal conditions, drought, LCC, different land uses and different degrees of drought aggravation or mitigation to changes in NPP. (1) The average NPP over the 15 years was generally low, with an average of approximately 227 gC/m^2^, and it decreased from southeast to northwest. The highest NPP value was found for forest land, followed by cropland, and the lowest was found for grassland. Overall, a linear increasing trend of NPP was observed for cropland and grassland, with the largest increasing value for cropland and the fastest increasing speed for grassland, and a linear decreasing trend of NPP was observed forest. (2) The influence of hydrothermal conditions on NPP was the strongest, followed by drought, and LCC was the lowest.The contribution to NPP decrease was slightly greater than that to NPP increase . (3) Approximately 61% of the NPP reduction was affected by the changes in hydrothermal conditions, ~33% was affected by drought, ~13% was affected by the aggravation of drought to light drought, and ~10% was affected by the aggravation of drought to medium drought; moreover, ~59% of the NPP increase was affected by the change in hydrothermal conditions, ~32% was affected by drought, ~12.7% was affected by the alleviation of light drought, and ~9.4% was affected by the alleviation of moderate drought. (4) The impact of LCC on NPP was unevenly distributed and generally accounted for between 5% and 10%, with ~1.4% of the NPP reduction affected by LCC. Conversion of cropland to grassland or non-NPP production land had the greatest impact on NPP reduction, with an average of approximately 0.3%; with ~0.5% of the NPP increase affected by LCC. Grassland conversion to cropland or forest land had the greatest impact on the increase in NPP, with an average of approximately 1.1%.

# Acknowledgments:

We appreciate the Institute of Geographical Sciences and Resources of the Chinese Academy of Sciences and NASA for freely providing the data.

# Author Contributions:

**Conceptualization**: Xiaowei Jiang, Jianjun Bai.

**Data Curation**: Xiaowei Jiang.

**Formal Analysis**: Xiaowei Jiang.

**Writing – Original Draft** Preparation: Xiaowei Jiang.

**Writing – Review & Editing**: Jianjun Bai.

# References：

1. Pavlov AA, Kasting JF, Brown LL, Rages KA, Freedman R. Greenhouse warming by CH4 in the atmosphere of early Earth. Journal of geophysical research. 2000;105(E5):11981-11990. doi: 10.1029/1999je001134. PubMed PMID: 11543544.
2. IPCC. Climate Change 2007—The Physical Science Basis: Contribution of Working Group I to the Fourth Assessment Report of the Intergovernmental Panel on Climate Change. Cambridge Univ.Press, New York.2007.
3. Sitch S, Huntingford C, Gedney N, Levy PE, Lomas M, Piao SL, et al. Evaluation of the terrestrial carbon cycle, future plant geography and climate-carbon cycle feedbacks using five Dynamic Global Vegetation Models (DGVMs). Global change biology. 2008;14(9):2015-2039. doi: 10.1111/j.1365-2486.2008.01626.x.
4. Fang JY. Forest productivity in China and its response to global changes. Acta phytoecologica sinica. 2000;24(5):513-517.
5. Liu G, Sun R, Xiao ZQ, Cui TX. Analysis of spatial and temporal variation of net primary productivity and climate controls in China from 2001 to 2014. Acta Ecologica Sinica. 2017;37(15):4936-4945. doi: 10.5846/stxb201604290822.
6. Cleveland CC ,Townsend AR, Taylor P, Alvarez-Clare S, Bustamante MMC, Chuyong G,et al. Relationships among net primary productivity, nutrients and climate in tropical rain forest: a pan-tropical analysis. Ecology letters. 2011;14(12):1313-1317. doi: 10.1111/j.1461-0248.2011.01711.
7. Peng D, Wu C, Zhang B, Huete A, Zhang X, Sun R, et al. The Influences of Drought and Land-Cover Conversion on Inter-Annual Variation of NPP in the Three-North Shelterbelt Program Zone of China Based on MODIS Data. PloS one. 2016;11(6):1-22. doi: 10.1371/journal.pone.0158173. PubMed PMID: 27348303; PubMed Central PMCID: PMC4922575.
8. Zhang L, Xiao J, Li J, Wang K, Lei L, Guo H. The 2010 spring drought reduced primary productivity in southwestern China. Environmental Research Letters. 2012;7(4):045706. doi: 10.1088/1748-9326/7/4/045706.
9. Zhao MS, Steven W. Drought–induced reduction in global terrestrial net primary production from 2000 through 2009. Science. 2010;329(5994):940-943.
10. Potter C, Klooster S, Hiatt C, Genovese V, Castilla-Rubio JC. Changes in the carbon cycle of Amazon ecosystems during the 2010 drought. Environmental Research Letters. 2011;6(3):034024. doi: 10.1088/1748-9326/6/3/034024.
11. Zhu YY, Han L, Zhao YH, Ao Y, Li JJ, Xiu KB, et al. Simulation and spatiotemporal analysis of vegetation NPP in northwest China. Chinese Journal of Ecology. 2019;38(6):1861-1871. doi: 10.13292/j.1000-4890.201906.012.
12. Cheng M, Wang RH, Xue HX, li Q. Effects of drought on ecosystem net primary production in northwestern China. Journal of Arid Land Resources and Environment. 2012;06(26):4-10. doi: 10.13448/j.cnki.jalre.2012.06.026.
13. Haberl H, Erb KH, Krausmann F, Loibl W, Schulz N, Weisz H. Changes in ecosystem processes induced by land use: Human appropriation of aboveground NPP and its influence on standing crop in Austria. Global Biogeochemical Cycles. 2001;15(4):929-942. doi: 10.1029/2000gb001280.
14. Li G, Hang GY, Wang Q, Wang XT, Gang ZN, Liu CZ. Spatial-temporal distribution of the ecological service value and NPP of cultivated land on Longdong Loess Plateau. Acta Prataculturae Sinica. 2011;20(6):18-25.
15. Zang XQ, Sun Y, Wang WF, Liu YY, Ren Y. Regional response of temperature change in the arid regions of China to global warming. Arid Zone Research. 2010;27(4):592-599. doi: 10.13866/j. azr.2010. 04.012.
16. Gang C, Zhao W, Zhao T, Zhang Y, Gao X, Wen Z. The impacts of land conversion and management measures on the grassland net primary productivity over the Loess Plateau, Northern China. The Science of the total environment. 2018;645:827-836. doi: 10.1016/j.scitotenv.2018.07.161. PubMed PMID: 30031340.
17. Su C, Fu B. Evolution of ecosystem services in the Chinese Loess Plateau under climatic and land use changes. Global and Planetary Change. 2013;101:119-128. doi: 10.1016/j.gloplacha.2012.12.014.
18. Prince SD, Goward SN. Global Primary Production: A Remote Sensing Approach. Journal of Biogeography. 1995;22(4/5):815-835.
19. Wang XM, Zhang CX, Hasi E, Dong ZB. Has the Three Norths Forest Shelterbelt Program solved the desertification and dust storm problems in arid and semiarid China? Journal of Arid Environments. 2010;74(1):13-22. doi: 10.1016/j.jaridenv.2009.08.001.
20. Noy-Meir I. Desert Ecosystems: Environment and Producers. Annual Review of Ecology and Systematics. 1973;4(1):25-51.
21. Guo Q, Hu Z, Li S, Li X, Sun X, Yu G. Spatial variations in aboveground net primary productivity along a climate gradient in Eurasian temperate grassland: effects of mean annual precipitation and its seasonal distribution. Global change biology. 2012;18(12):3624-3631. doi: 10.1111/gcb.12010.
22. Vicente-Serrano S M, Beguería S, López-Moreno, Juan I. A Multiscalar Drought Index Sensitive to Global Warming: The Standardized Precipitation Evapotranspiration Index. Journal of Climate. 2010;23(7):1696-1718. doi: 10.1175/2009JCLI2909.1.
23. Jiang XW, Bai JJ, Liu XF. Research progress and prospect of integrated drought monitoring based on multisource information. Advances in Earth Science. 2019;34(3):275-287. doi: 10.11867/j.issn.1001-8166.2019.03.0275.
24. Vicente-Serrano SM. Differences in Spatial Patterns of Drought on Different Time Scales: An Analysis of the Iberian Peninsula. Water Resources Management. 2006;20(1):37-60. doi: 10.1007/s11269-006-2974-8.
25. Piao S, Ciais P, Friedlingstein P, Noblet-Ducoudré DND, Patricia C, Nicolas V, et al. Spatiotemporal patterns of terrestrial carbon cycle during the 20th century. Global Biogeochemical Cycles. 2009;23(4).
26. Cai S, Liu D, Sulla-Menashe D, Friedl MA. Enhancing MODIS land cover product with a spatial–temporal modeling algorithm. Remote Sensing of Environment. 2014;147:243-255. doi: 10.1016/j.rse.2014.03.012.
27. Liang L, Gong P. An assessment of MODIS Collection 5 global land cover product for biological conservation studies. 2010 18th International Conference on Geoinformatics IEEE. 2010. doi: 10.1109/GEOINFORMATICS.2010.5567991.
28. Zhang LX, Zhou DC, Fan JW, Hu ZM. Comparison of four light use efficiency models for estimating terrestrial gross primary production. Ecological Modelling. 2015;300:30-39. doi: 10.1016/j.ecolmodel.2015.01.001.
29. Cao M, Prince SD, Small J, Goetz SJ. Remotely Sensed Interannual Variations and Trends in Terrestrial Net Primary Productivity 1981–2000. Ecosystems. 2004;7(3):233-242. doi: 10.1007/s10021-003-0189-x.
30. Xiao X, Zhang Q, Saleska S, Hutyra L, De Camargo P, Wofsy S, et al. Satellite-based modeling of gross primary production in a seasonally moist tropical evergreen forest. Remote Sensing of Environment. 2005;94(1):105-122. doi: 10.1016/j.rse.2004.08.015.
31. Nepstad DC, Moutinho P, Dias-Filho MB, Davidson E, Cardinot R, Markewitz D, et al. The effects of partial throughfall exclusion on canopy processes, aboveground production, and biogeochemistry of an Amazon forest. Journal of Geophysical Research: Atmospheres. 2002.
32. Chi Y, Shi HH, Sun JK, Li J, Yan F, Fu ZY. Spatio-temporal characteristics and main influencing factors of vegetation net primary productivity in the Yellow River Delta in recent 30 years. Acta Ecologica Sinica. 2018;38(8):2683-2697. doi: 10.5846/stxb201705301000.
33. Liu XJ, Zho J, Du ZQ, Zhang H. Net primary productivity pattern of grassland in China and its relationship with hydrothermal factors during 1993-2015. Bulletin of Soil and Water Conservation. 2018;38(1):299-305.
34. Pederson GT, Gray ST, Fagre DB, Graumlich LJ. Long-Duration Drought Variability and Impacts on Ecosystem Services: A Case Study from Glacier National Park, Montana. Earth Interactions. 2006;10(4):1-28. doi: 10.1175/ei153.1.
35. Zhao A, Zhang A, Lu C, Wang D, Wang H, Liu H. Spatiotemporal variation of vegetation coverage before and after implementation of Grain for Green Program in Loess Plateau, China. Ecological Engineering. 2017;104:13-22. doi: 10.1016/j.ecoleng.2017.03.013.
36. XiuY. Evaluation on ecological benefits of returning farmland to forest in Baoji city. Bulletin of Soil and Water Conservation. 2017;37(6):248-255.
