# Supplementary material for: Quantifying the impacts of drought and ecological restoration on net primary production changes in the Chinese Loess Plateau
Source: PLoS One. 2020 Sep 24;15(9):e0238997. doi: 10.1371/journal.pone.0238997 (PMC7514050; doi:10.1371/journal.pone.0238997)
Supplement: S1 Data — (DOCX) [file pone.0238997.s002.docx]

Data availability description

Relevant data underlying the findings described in manuscript and the minimal data set necessary to replicate our study’s findings is located in our manuscript (The minimum dataset needed to replicate our results mainly includes NPP, MODIS, and SPEI data). In addition, the NPP, MODIS, and SPEI data is third-party data, and the authors had no special access privileges to the data and that other researchers will be able to access the data in the same manner as the authors. They can be obtained in the following ways:

1. NPP product is available from http://www.resdc.cn/Default.aspx.

2. MODIS land cover product is available from https://ladsweb.modaps.eosdis.nasa.gov.

3. SPEI product is available from http://digital.csic.es.
